# HappyTools: A software for high-throughput HPLC data processing and quantitation

**DOI:** 10.1371/journal.pone.0200280

**Published:** 2018-07-06

**Authors:** Bas Cornelis Jansen, Lise Hafkenscheid, Albert Bondt, Richard Andrew Gardner, Jenifer Lynn Hendel, Manfred Wuhrer, Daniel Ian Richard Spencer

**Affiliations:** 1 Research & Development, Ludger Limited., Abingdon, Oxfordshire, United Kingdom; 2 Department of Rheumatology, Leiden University Medical Center, Leiden, Zuid-Holland, The Netherlands; 3 Center for Proteomics and Metabolomics, Leiden University Medical Center, Leiden, Zuid-Holland, The Netherlands; Swiss Institute of Bioinformatics, SWITZERLAND

## Abstract

High-performance liquid chromatography (HPLC) is widely used for absolute quantitation. The advent of new columns and HPLC technology has enabled higher sample throughput, and hence, larger scale studies that perform quantitation on different sample types (*e*.*g*. healthy controls *vs*. patients with rheumatoid arthritis) using HPLC are becoming feasible. However, there remains a lack of methods that can analyse the increased number of HPLC samples. To address this in part, the modular toolkit HappyTools has been developed for the high-throughput targeted quantitation of HPLC measurements. HappyTools enables the user to create an automated workflow that includes retention time (t_r_) calibration, data extraction and the calculation of several quality criteria for data curation. HappyTools has been tested on a biopharmaceutical standard and previously published clinical samples. The results show comparable accuracy between HappyTools, Waters Empower and ThermoFisher Chromeleon. However, HappyTools offered superior precision and throughput when compared with Waters Empower and ThermoFisher Chromeleon. HappyTools is released under the Apache 2.0 license, both the source code and a Windows binary can be freely downloaded from https://github.com/Tarskin/HappyTools.

## Introduction

High-performance liquid chromatography (HPLC) with fluorescence detection (FD) is a widely used technique for the analysis of biological samples, *e*.*g*. proteins, metabolites and glycans. Glycans are a class of post-translational modifications that can be added to a protein, thereby modifying the structure and function of the protein [1,2]. One of the main advantages of HPLC-FD in glycan analysis is its ability to separate isomers, *e*.*g*. enabling the differentiation of the two isomers of one of the main glycans of immunoglobulin G (IgG) [3]. Furthermore, the relatively low cost of an HPLC-FD instrument compared to a matrix-assisted laser desorption/ionization (MALDI)-time of flight (TOF)-mass spectrometry (MS) or liquid chromatography (LC)-electrospray ionization (ESI)-MS setups means that an HPLC-FD setup is very cost-effective for profiling biological and biopharmaceutical samples. Traditionally, sample analysis on the HPLC is time consuming due to gradient lengths. However, in recent years column manufacturers and researchers have been working on improving the throughput of HPLC [4–6]. Therefore, the bottleneck is slowly shifting from the experimental side (*e*.*g*. glycan release and sample clean-up) to the data analysis side. Consequently, many HPLC-FD studies use a limited number of samples and tend to rely on commercially available software packages for the data analysis, *e*.*g*. Waters Empower or ThermoFisher Chromeleon [7,8].

There are several software tools available to assist with the identification and annotation of HPLC-FD data, such as GlycoDigest and GlycoStore [9–11]. However, despite the widespread availability of HPLC-FD equipment there has been a lack of novel quantitative software tools for data processing being developed compared to other equipment, such as MALDI-TOF-MS or LC-ESI-MS [12–14]. Such novel software tools have enabled the largest glycomics study to date, a study comprising the acquisition and comparison of over 20,000 MALDI-Fourier-transform ion cyclotron resonance (FTICR)-MS spectra [15]. The lack of such freely available high-throughput (HT) data processing tools for HPLC-FD has hindered its application in large scale studies. To enable larger scale studies, a software tool dedicated to the data processing of HPLC-FD data should contain retention time (t_r_) calibration, peak detection, peak quantitation and should facilitate data curation.

To address the deficiency of software tools for HPLC-FD data processing, a modular toolkit called HappyTools has been developed that can perform all the steps of a completely automated data analysis workflow, including automated peak detection, t_r_ calibration and peak quantitation. To facilitate rapid development and reduce the overall source code complexity, HappyTools uses standardized source code from our previous work [13,14]. Key improvements include automated peak detection, a Gaussian Peak Quality (GPQ) score and a plugin functionality. The availability of automated peak detection addresses a problem that was present in our previous work, namely that a pre-defined analyte list is required to quantify a sample [13,14]. The automatically determined analyte list significantly reduces the time required to generate the final analyte list for quantitation. The GPQ is part of several HPLC-FD specific quality control (QC) criteria that HappyTools can calculate to facilitate data curation. It is calculated as the percentage of total peak area (A_p_) that is explained by a fitted Gaussian peak, *i*.*e*. total A_p_ divided by the Gaussian A_p_. The GPQ parameter can help to identify overlapping peaks, peak tailing and peak fronting. The current features of the HappyTools toolkit should facilitate most HPLC-FD based research, however some studies will require highly customized features. Therefore, a plugin system was developed for HappyTools that enables any externally developed Python package to utilize all the functions of HappyTools. Key benefits of this approach are that the development time for a custom feature is significantly reduced and that the developers only have to maintain their own codebase as the HappyTools codebase will be maintained by the HappyTools development team. For example, an affiliated group is currently developing a highly specialized plugin for automated peak detection algorithms of specific sample types [16].

While HappyTools can be used on any 2D chromatographic or electrophoretic data, it was developed and tested using chromatographic glycomics data. Specifically, the performance and applicability of HappyTools has been demonstrated on two sample sets, namely a set of 9 replicates of a biopharmaceutical monoclonal antibody (mAb) reference standard and a set of previously published anti-citrullinated protein antibodies (ACPA)-IgG samples [17]. The results show that HappyTools enabled a highly automated data processing workflow that yielded comparable accuracy but improved precision and throughput when compared to either Waters Empower or ThermoFisher Chromeleon.

## Design and implementation

### General design and dependencies

HappyTools has been developed as a graphical application for Python 2.7 [18]. The program requires several external Python libraries to function, *i*.*e*. SciPy, numpy and matplotlib [19–21]. The GitHub repository for HappyTools contains a pip freeze output, to facilitate the deployment of the package. A Windows binary of HappyTools is also available for each release on the GitHub repository. A modular design was used to facilitate further development and to enable the use of some of the HappyTools functions in other programs. For example, Python plugins can be added to HappyTools by placing them in the plugin folder, which includes an example called “Demo”. The source code and all related HappyTools files have been released under the Apache 2.0 License [22].

### Data import

HappyTools is designed to use non-proprietary data formats, *e*.*g*. a text format that can be exported from ThermoFisher Chromeleon. The currently supported formats are ThermoFisher Chromeleon “.txt” and Waters Empower “.arw” files. HappyTools will implement support for additional data formats in future releases, until all current data formats are supported.

### Automated peak detection

HappyTools performs t_r_ calibration and quantitation based on a pre-defined peak list, which is a tab separated text file with the peak name, peak t_r_ and the t_r_ window (Δt_r_) per line. A peak file can either be provided by the user or generated by the automated peak detection algorithm of HappyTools. Many methods exist for automated peak detection in HPLC, ranging from using the 1^st^ and 2^nd^ order derivative of locally smoothed signals to bi-Gaussian mixture models [16,23,24]. HappyTools uses an approach that uses the local maxima and minima of a 1^st^ order derivative of the piecewise polynomial fitted raw data, which we have dubbed the first order derivative—Gaussian peak detection (FOD-GPD) algorithm. First, the overall background and noise are determined using a modification of the MassyTools method, *i*.*e*. taking the average and standard deviation of a set of sequential data points that yields the lowest average [13]. Subsequently, the algorithm fits a univariate spline through a user-defined region of the raw data. The 1^st^ order derivative (f’(x)) of the univariate spline is determined and used to identify the local maxima and minima of f’(x), as this offered superior performance over identifying where the second order derivative (f”(x)) is 0. The highest intensity data point that falls between a neighbouring maximum and minimum of f’(x) is used to determine the intensity cut-off for the subsequent loop. Specifically, a setting of 1% tells the next step of the algorithm to continue until the current highest intensity data point falls below 1% of the initial highest data point.

**Fig 1:**
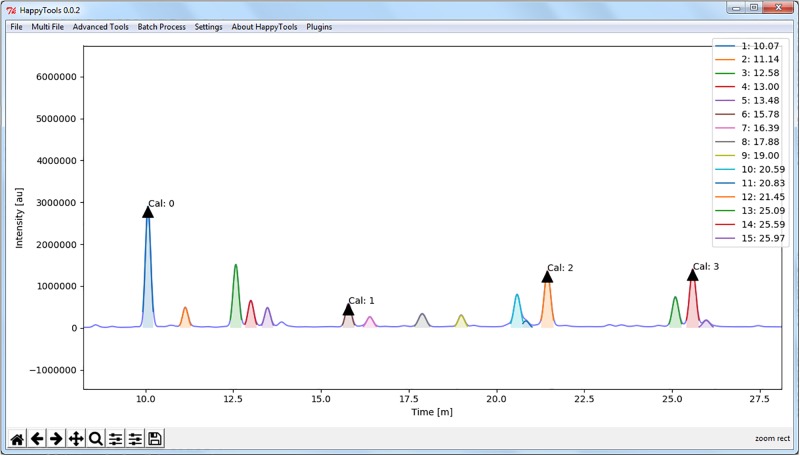
Automated peak detection using HappyTools. An HPLC measurement of 2-aa ACPA-IgG glycans was used to perform automated peak detection, with a threshold of 5% relative abundance of the initial main peak at around 10 min. Automated peak detection using a method that fits Gaussian peaks identified 15 peaks, which includes a peak that partially overlaps with another peak at 20.85 min. However, it is important to note that using a Gaussian function on non-Gaussian data that is the result of chromatographic problems will identify additional peaks (S2 Fig).

The main part of the algorithm is a loop that continues until the highest intensity falls below the user-specified cut-off value. A new univariate spline is fitted for each iteration of the loop, from which the local maxima and minima are determined by using the 1^st^ order derivative. The data points between a neighbouring maximum and minimum of f’(x) that yield the highest intensity data point in f(x) are used to fit a Gaussian peak. The last step in the loop is to subtract the Gaussian peak from the overall data. A schematic representation of the algorithm is presented in S1 Fig. The FOD-GPD algorithm will show the raw data and the automatically detected peaks in the graphical user interface (GUI) of the program for the user to review (Fig 1). The automatically detected peaks can then be stored using the HappyTools peak list format, where each line contains an arbitrary peak number, the t_r_ of each Gaussian peak and a t_r_ window based on the full width at half maximum (FWHM). Furthermore, a set of potential calibrant peaks is automatically determined by taking a set of the four highest intensity data points that the algorithm attempts to space evenly in t_r_.

### Retention time calibration

High quality quantitation requires that all peaks for quantitation are well defined. Therefore, t_r_ calibration of chromatograms is an essential step in HPLC data processing. A set of t_r_ calibrated chromatograms enables the consistent quantitation of a low abundant analyte that is not present in all chromatograms. Furthermore, a stringent t_r_ calibration also enables integration using the minimum Δt_r_ to match the actual peak width. Whereas, a non-stringent t_r_ calibration forces the Δt_r_ to be set wider than the actual peak width. HappyTools performs t_r_ calibration based on a user defined calibrant peak list instead of the commonly used alignment based on automatically detected features [25]. The format of the calibrant peak list is similar to the quantitation peak list, specifically it is a tab separated text file with the peak name, t_r_ and Δt_r_. The algorithm takes the highest intensity data point within each calibrant peak t_r_ ± Δt_r_ as the observed t_r_. The signal-to-noise ratio (S/N) of each calibrant peak is then calculated, and only calibrant peaks passing a user specified S/N threshold are retained. A 2^nd^ degree polynomial (f_p_(x)) is fitted through the retained t_r_ coordinates (t_r_(Obs.), t_r_(Exp.)), only if the number of retained t_r_ coordinates surpasses the user defined minimum number of calibrants. Finally, the t_r_ calibration is performed using the formula t_r_(new) = f_p_(t_r_(original)).

### Peak quantitation

There are several methods to quantify a peak, a commonly utilised technique is to fit a peak shape on the raw data and report the area of the fitted peak shape [24,26]. This method works well for high abundant analytes that generally show symmetric peak shapes. However, for low abundant analytes that may not have a clear peak shape the peak fitting method yields poor results [14]. An alternative method to determine the A_p_ is peak integration of the raw data as follows:

$${Α}_{p}=\sum_{i=1}^{n}t_{m}I_{i}$$ (1)

where t_m_ is the time that it takes to measure a single data point and I_1_, I_2_ … I_n_ are the signal intensities. Recent research using LC-MS data has shown that peak integration provides comparable results for high abundant analytes and yields superior results for low abundant analytes using MALDI-TOF-MS data [13]. Therefore, HappyTools performs peak quantitation using an adaptation of the peak integration method previously used in both MassyTools and LaCyTools (Eq 1) [13,14].

### Quality control criteria

HappyTools calculates three QC criteria for each analyte to facilitate data curation. The first criterion calculates the residual retention time (r_t_) by comparing the expected and observed t_r_ of each signal. The observed t_r_ is determined by taking the function maximum of a fitted interpolated univariate spline through all the data points of a signal. The r_t_ is then calculated for each analyte as described below (Eq 2).

$$r_{t}=|t_{r}(Obs.)-t_{r}(Exp.)|$$ (2)

HappyTools can also determine the S/N of an analyte, based on a implementation used in both MassyTools and LaCyTools [13,14]. Briefly, the t_r_ region around an analyte that has the lowest average intensity is identified, from which the average intensity is used as background and the standard deviation is used as noise. Subsequently, the background is subtracted from the maximum intensity prior to dividing the remainder by the noise. GPQ is the third criterion offered by HappyTools, which determines how well a single Gaussian peak matches the quantified signal [27]. The background value is subtracted from each data point, after which a Gaussian is fitted to all background subtracted data points. The GPQ is then calculated by dividing the A_p_ of the fitted Gaussian peak with the background subtracted A_p_ (S3 Fig).

## Materials and methods

### Samples

This study used previously measured and published clinical samples, to assess the performance of HappyTools [17]. Permission for conduct of the study was in compliance with the Helsinki Declaration, and was approved by the Ethics Review Board at the Leiden University Medical Center.

### Chemicals, reagents and enzymes

Immunoglobulin G 1 from human myeloma (I5154) and acetic acid were acquired from Sigma-Aldrich (Dorset, UK). Trypsin Gold (V5280) was obtained from Promega (Madison, USA). Phosphate-buffered saline (PBS), trifluoroacetic acid (TFA) and hydrophilic interaction amide cartridges (LudgerClean LC-A-24) are all components in the LudgerTagTM V-tag Glycopeptide Labeling and Enrichment Kit (LT-VTAG-24) which is sourced from Ludger Ltd (Abingdon, UK). Acetonitrile was acquired from Romil (Cambridge, UK).

### Sample preparation

A total of 10 μg of IgG was digested using trypsin Gold. Samples were buffer exchanged into PBS to a concentration of 1 mg/mL. 10 μL of the protein solution was transferred into an Eppendorf vial and 4 μL of 0.5 mg/mL trypsin in 0.1 M acetic acid in water was added. The mixture was incubated for 1 h at 55°C with sonication. The sample pots were cooled to room temperature and 5 μL of the V-Tag labelling reagent was added directly to each digested sample. The samples were vortexed and briefly centrifuged. The labelling reaction was allowed to proceed for 1 hour at 37 °C. The V-Tag labelled samples were cleaned up using hydrophilic interaction amide LC-A cartridges. Each sample was loaded onto a primed cartridge in 76% aqueous acetonitrile. The cartridge was washed with 76% acetonitrile, 0.1% TFA in water solution. The purified V-Tag labelled glycopeptides were eluted from the amide cartridge in 0.5 mL of a solution containing 40% ACN and 0.1% TFA in water.

### Data acquisition

V-Tag labelled samples were analysed by HILIC-UPLC using an ACQUITY UPLC^®^ BEH-Glycan 1.7 μm, 2.1 x 150 mm column from Waters at 40 °C on an ACQUITY UPLC H Class instrument with a fluorescence detector (λ_ex_ = 250 nm, λ_em_ = 360 nm), controlled by Empower version 3.0 build 3471. The solvents used for the HILIC-UPLC were 50 mM ammonium formate (A) made from Ludger stock buffer LS-BUFAMMFORM-2M-50ML (Ludger) and Acetonitrile 190 SpS (B). The gradient used for HILIC-UPLC was run as follows: 0.0 min, 28% solvent A; 54.0 min, 48% solvent A; 57.0 min, 100% solvent A; 60 min. 28% solvent A. The flow rate was 0.4mL/min from 0.0 to 57.min, from 57.0 to 60.0 min the flow rate was 0.2mL/min.

### Data processing

V-Tag labelled samples were processed using Waters Empower, ThermoFisher Chromeleon and HappyTools. The Waters Empower processing included automated peak detection and integration, using the ApexTrack algorithm. The ThermoFisher Chromeleon processing of the V-Tag labelled glycopeptides included automated peak detection and integration, using the default method. ThermoFisher Chromeleon could not reliably quantify two peaks that were partially overlapping, therefore these two peaks were quantified as a single peak. The results were exported to excel, with each measurement producing a single excel file from which the absolute A_p_s were taken to calculate the average relative A_p_ and coefficient of variation (CV).

V-Tag labelled glycopeptide samples were first t_r_ calibrated using HappyTools using four calibrant peaks (S1 Table). The mean t_r_ for all seven glycopeptide peaks was acquired by overlaying the calibrated chromatograms using HappyTools’ normalized batch plot functionality. HappyTools was used to quantify all parameters for the seven glycopeptide peaks (S2 Table). The following settings were used for HappyTools; start t_r_: 11.0 min, end t_r_: 25.0 min, background window: 1.0 min, minimum number of peaks for calibration: 4, minimum signal-to-noise for calibration: 9, minimum relative abundance for peak detection: 1%, order of baseline function: 1 and number of data points for the determination of the baseline: 100.

HappyTools was also used to process a set of previously published ACPA-IgG samples that were previously processed using ThermoFisher Chromeleon [17]. HappyTools was run without t_r_ calibration as the measurements were already calibrated. Subsequently, the quantitation was performed using the same peaks as were used in the original study with a quantitation width of 0.2 min. The following settings were used for HappyTools; start t_r_: 5.0 min, end t_r_: 30.0 min (ACPA-IgG Fab and ACPA-IgG part 2) or 35.0 min (ACPA-IgG part 1), background window: 1.0 min, minimum number of peaks for calibration: 4, minimum signal-to-noise for calibration: 27 (ACPA-IgG part 1 and ACPA-IgG part 2) or 9 (ACPA-IgG Fab), order of baseline function: 1 and number of data points for the determination of the baseline: 100.

## Results

HappyTools was tested using data from a biopharmaceutical standard and previously published clinical samples to test the accuracy, precision and throughput of the quantitation. The accuracy achieved by HappyTools is comparable to both Waters Empower and ThermoFisher Chromeleon, while the precision of HappyTools was superior to both Waters Empower and ThermoFisher Chromeleon. Furthermore, the processing time using HappyTools was significantly shorter than the total processing time using both Waters Empower and ThermoFisher Chromeleon. The complete datasets, a visual tutorial of HappyTools and a document describing how to reproduce the results used in this study have been included as supplementary material (S1 Data). The results below demonstrate the application of HappyTools on relatively simple samples. Notably, preliminary data on more complex samples, *e*.*g*. full plasma *N*-glycome, are promising and indicate a broad applicability of the tool (data not shown).

### Biopharmaceutical mAb reference standard

**Fig 2:**
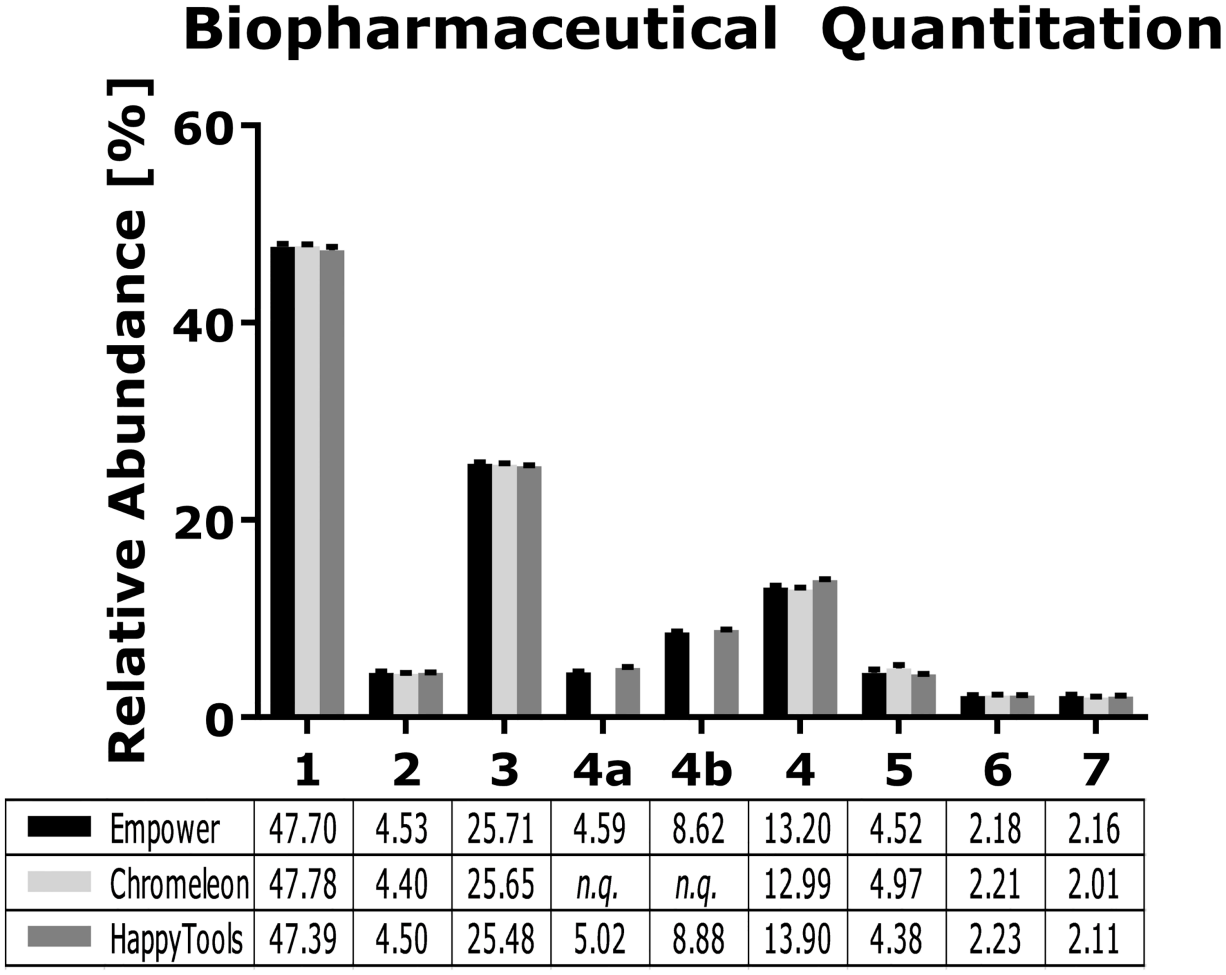
Biopharmaceutical quantitation using Waters Empower, ThermoFisher Chromeleon and HappyTools. A set of 9 replicates of V-Tag labelled tryptic glycopeptides were used to compare the three different software tools. The results show that all methods yield comparable accuracy, while HappyTools yields superior precision. Peak 4a and peak 4b could not be quantified separately using ThermoFisher Chromeleon but was instead quantified as a singular peak. The individual values for peaks 4a and peak 4b obtained from Waters Empower and HappyTools were summed to compare with ThermoFisher Chromeleon.

A set of 9 replicates of V-Tag labelled tryptic glycopeptides from a mAb reference standard was measured by hydrophilic interaction liquid (HILIC) chromatography and processed using Waters Empower, ThermoFisher Chromeleon and HappyTools. HappyTools’ automated peak identification was used to detect all peaks above 1% relative abundance of the initially highest peak, which yielded 20 peaks (S4 Fig). The number of peaks that were used for the comparison was reduced to 7 to match the number of peaks that could be quantified using both Waters Empower and ThermoFisher Chromeleon. The results show that the relative A_p_s reported by HappyTools are comparable to both Waters Empower and ThermoFisher Chromeleon (Fig 2 and S3 Table). The precision of HappyTools was superior to both Waters Empower and ThermoFisher Chromeleon, with all peaks except one showing a lower CV (S3 Table). HappyTools showed an average 2.22- and 2.26-fold change improvement of the CVs when compared to Waters Empower and ThermoFisher Chromeleon, respectively (S4 Table). It is possible to further improve the accuracy and precision by adjusting the quantitation window per glycopeptide, *e*.*g*. lowering the quantitation window for all analytes from ± 0.15 min to ± 0.10 min lowered the CV from 0.67% to 0.53% for the most abundant glycopeptide (data not shown).

Lastly, the throughput of HappyTools was superior to both Waters Empower and ThermoFisher Chromeleon. The total processing time using both ThermoFisher Chromeleon and Waters Empower included manual adjustment of the peak edges within each run, to ensure that the results were comparable. The average relative abundance and variation were acquired by either processing the individual results in Excel (ThermoFisher Chromeleon) or by creating several templates (Waters Empower). The processing time using HappyTools included performing automated peak detection and manual curation of the automatically detected peaks. The resulting total processing time using HappyTools was 1 hour, whereas the total processing time using ThermoFisher Chromeleon or Waters Empower was 3 hours.

### Clinical samples

**Fig 3:**
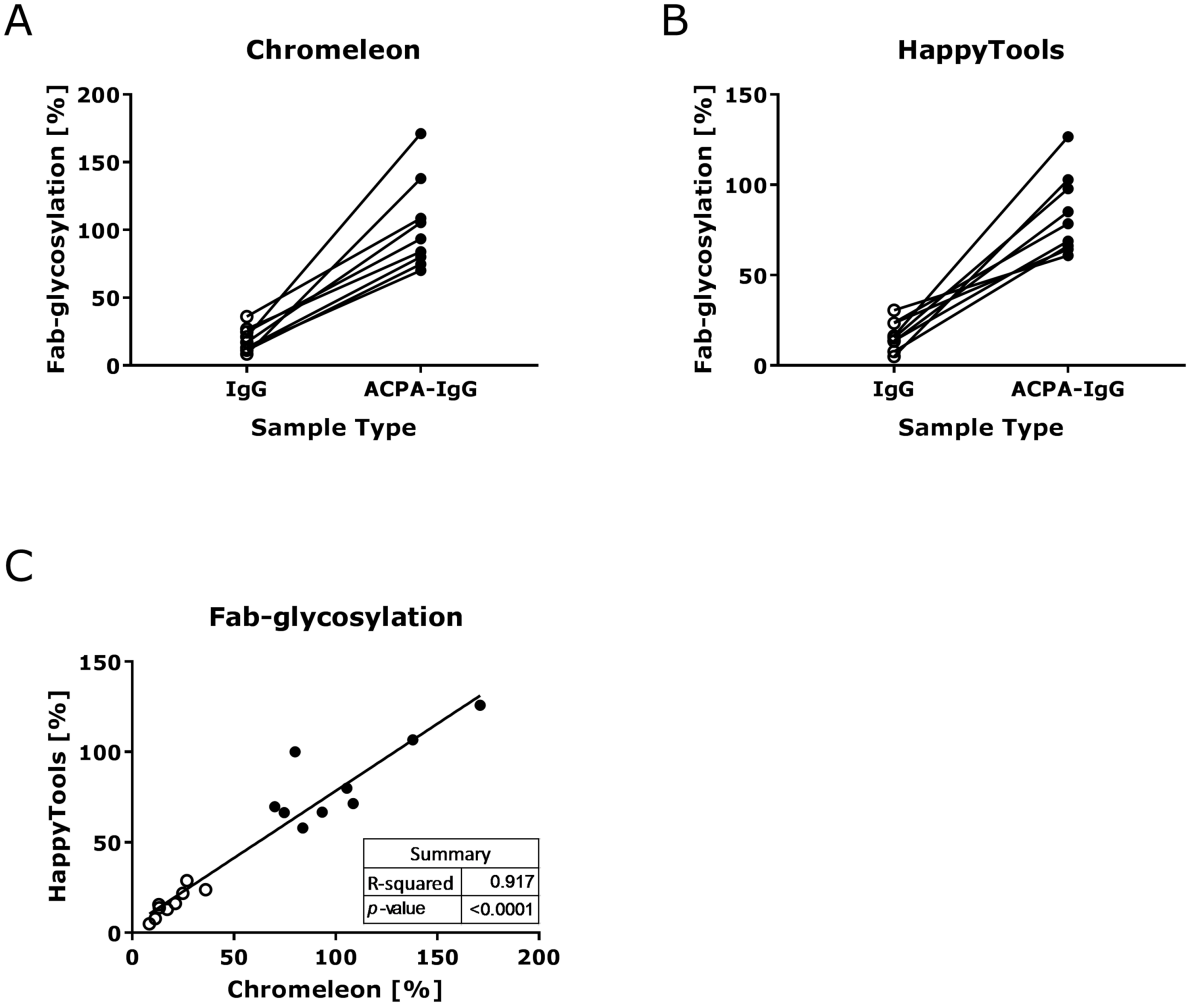
ThermoFisher Chromeleon vs. HappyTools comparison of total IgG and ACPA-IgG. A total of 36 UHPLC measurements was used to compare the quantitation as performed by HappyTools with the original quantitation performed using ThermoFisher Chromeleon. Data points that derived from IgG are indicated by an open circle, while data points that derive from ACPA-IgG are portrayed by closed circles. The results show that both programs yield a similar result and more importantly that there is a significant (p < 0.0001) correlation between the two data sets.

A total of 36 measurements of 2-aminobenzoic acid labelled ACPA-IgG, ACPA-IgG Fc and ACPA-IgG Fab glycans were previously prepared, measured using HILIC-ultra high performance liquid chromatography (UHPLC) and exported to .txt format using ThermoFisher Chromeleon [17]. The ACPA-IgG and ACPA-IgG Fab measurements were used to assess if HappyTools produces comparable results to the previously used ThermoFisher Chromeleon in a clinical setting. Specifically, Fab glycosylation in IgG has been found to be vastly different between ACPA-IgG and normal IgG, with ACPA-IgG showing on average five times higher levels of Fab glycosylation [17]. The observed difference in Fab glycosylation suggests that ACPA-IgG may mediate novel immunological activities [17]. The study compared IgG and ACPA-IgG Fab glycosylation (S5 Fig), hereby the glycans of the F(ab’)_2_ fragments and Fc glycopeptides were compared to the glycan profile of the total antibody and the percentage of Fab glycosylation was calculated. The following formula was used to calculate the percentage Fab-glycosylation:

$${Glycosylation}_{Fab}[\%]=\frac{\left(\frac{{G2S2}_{Total}}{{G2S2}_{Fab}}\right)}{\left(\frac{{G1F}_{Total}}{{G1F}_{Fc}}\right)}$$ (3)

where G2S2 consists of GP21, GP22, GP23 and GP24, G1F_Total_ consists of GP8 and GP9 and G1F_Fc_ is taken from glycopeptide measurements of the original publication [17]. The results for both Chromeleon and HappyTools show a higher percentage of Fab-glycosylation in ACPA samples than IgG samples, with the values reported by ThermoFisher Chromeleon and HappyTools showing a significant correlation (Fig 3 and S5–S7 Tables). This result shows that the same clinical finding, namely an increase in Fab-glycosylation in ACPA-IgG samples, can be observed with either ThermoFisher Chromeleon or HappyTools. Finally, the total throughput using HappyTools was far superior than what was achieved during the original study. Specifically, during the original study the processing took around 10 hours while the re-analysis using HappyTools took only 1 hour.

## Discussion

High performance liquid chromatography (HPLC) has long been considered the gold standard for quantitation of carbohydrates, specifically when combined with HILIC and 2-aminobenzamide (2-AB)-labelled glycans [28]. While the number of samples that is measured by HPLC has been increasing, *e*.*g*. a study into the Immunoglobulin G glycome measured 2298 individuals, the data analysis is mostly achieved using a high amount of manual processing using the manufacturers software [3]. Therefore, the main goal of HappyTools was to provide a framework independent of the manufacturer that would enable high-throughput processing of HPLC data, which includes t_r_ calibration, quantitation and the determination of various quality criteria. The application of the software on biopharmaceutical and clinical samples showed similar or better performance than either Waters Empower or ThermoFisher Chromeleon. The main improvements were observed in the precision and throughput. Specifically, the biopharmaceutical samples showed a 2.22- or 2.26-fold change improvement when compared to Waters Empower and ThermoFisher Chromeleon, respectively. Furthermore, the total processing time showed a 10-fold reduction for the biopharmaceutical samples and a 3-fold reduction for the clinical samples.

However, HappyTools was primarily designed as a targeted data processing package and therefore requires knowledge of the analytes that can be present in each sample. For instance, when comparing samples from a healthy and an immunocompromised source, the software requires a list of analytes for quantitation. To address this issue HappyTools includes a basic automated peak detection functionality, which identifies the t_r_ and Δt_r_ of all signals above a user-defined threshold. The implemented peak detection algorithm assumes that signals give a Gaussian peak shape. However, the algorithm will have difficulties with chromatograms that contain significant peak tailing. Therefore, it is important to curate the results when an automatically detected peak list is used for quantitation. HappyTools can also calculate several quality criteria that facilitate easy results curation, such as the Gaussian Peak Quality (GPQ) and the signal-to-noise (S/N) value. For example, the GPQ will yield a poor value for the tail of a peak if it is quantified based on an automatically determined peak list.

There are also some limitations to HappyTools, which include a lack of good manufacturing practice (GMP) functionality and the dependency on Python. The manufacturer software packages generally include options to protect data integrity and prevent data manipulation [29,30]. HappyTools currently does not contain any method to guarantee data integrity, *e*.*g*. by verifying if results match with a given data set. However, there are plans to include such a functionality by calculating a number representing the raw data (checksum) for each processed chromatogram and including it with the results. Furthermore, HappyTools will then also include the option to calculate the checksum for any chromatogram which will allow the researcher to validate that the results match the data set. The second limitation is that HappyTools requires Python 2.7 with several external libraries meaning that it requires some IT knowledge to deploy and use HappyTools. Therefore, a Windows binary will also be released for every major version/release of HappyTools which should enable researchers to easily try the software. Furthermore, we are also in the process of developing a web application-based version of HappyTools, which will allow researchers to perform automated t_r_ calibration, quantitation and the calculation of chromatographic quality criteria on a limited number of samples.

In summary, HappyTools provides a fully open-source and transparent toolkit for the high throughput data processing of HPLC data. HappyTools enables t_r_ calibration, quantitation and the calculation of various quality criteria. HappyTools has been shown to offer similar precision and superior throughput when compared to currently available software such as ThermoFisher Chromeleon. The source code for HappyTools and a Windows binary can be freely downloaded from www.github.com/Tarskin/HappyTools.

## Supporting information

S1 Fig HappyTools peak detection algorithm. The algorithm first creates a subset of the data, based on the user specified region of interest to ensure that artefacts are not examined by the algorithm. Subsequently, the background and noise are determined which will be used as a baseline for later Gaussian fitting steps. The 1^st^ order derivative is determined of a univariate spline that has been fitted to the data subset. The borders for each peak in the chromatogram is then determined by derivatizing the univariate spline and identifying where the local maxima and minima are of f’(x). The highest intensity data point of all peaks in the user specified region of interest is used to determine the intensity cut-off (e.g. 1% of the initial highest intensity). The main part of the algorithm is then repeated until the highest intensity data point is no longer above the intensity cut-off, and within each loop the borders of all remaining peaks are first determined by using a new univariate spline and it’s derivative. Subsequently, a Gaussian is fitted to the data that yields the highest intensity data point, after which the Gaussian is subtracted from the data.
(TIFF)

S2 Fig Gaussian peak fitting and non-Gaussian data. HappyTools uses a Gaussian function to identify chromatographic peaks, which can result in a single non-Gaussian peak being resolved as multiple peaks. (**A**) Two partially overlapping that can be confidently resolved using HappyTools, (**B**) A non-Gaussian peak or two partially overlapping Gaussian peaks, which is resolved as two separate peaks by HappyTools. These images were taken directly from HappyTools, after disabling the legend.
(TIFF)

S3 Fig Gaussian peak fitting on experimental data. This figure illustrates how the raw data is used to fit both a univariate spline and a Gaussian peak. The univariate spline is used to determine the centre of the experimental peak, which is used to determine the signal-to-noise ratio. The Gaussian fit is used to determine how much of the experimental peak area can be explained by an underlying Gaussian peak, which is the Gaussian peak Quality (GPQ).
(PDF)

S4 Fig Automated peak identification using V-Tag labelled tryptic glycopeptides. A total of 20 peaks was detected using HappyTools’ peak detection functionality between 10.0 and 30.0 min using a peak detection threshold of 1%. The displayed peak width was selected to be 2σ. However, several of the detected peaks are caused by either overlapping peaks or non-Gaussian peak shapes. Manual curation of the automatically detected peaks reduces the number to 13–15.
(PDF)

S5 Fig Immunoglobulin G (IgG) fragment antigen-binding (Fab) and anti-citrullinated protein antibodies (ACPA)-IgG chromatograms of a single patient. (**A**) IgG chromatogram, (**B**) IgG-Fab chromatogram, (**C**) ACPA-IgG chromatogram and (**D**) ACPA-IgG Fab chromatogram of patient 4. All chromatograms have been normalised to the highest peak between 10 and 60 minutes. The chromatograms have been plotted using the ‘Normalized Batch Plot’ functionality of HappyTools. The displayed glycan structures are based on the original publication that first measured and described these samples [17].
(PDF)

S1 Table V-Tag labelled tryptic glycopeptides peaks used for t_r_ calibration. Four glycopeptide peaks that were used to perform t_r_ calibration have been listed below, included are the peak name, peak t_r_ and peak Δt_r_.
(XLSX)

S2 Table V-TAG labelled tryptic glycopeptide peaks used for quantitation. All glycopeptide peaks that were used for quantitation are listed below, the table lists the peak name, the t_r_ and Δt_r_.
(XLSX)

S3 Table Quantitation comparison between Waters Empower, ThermoFisher Chromeleon and HappyTools using V-TAG labelled tryptic glycopeptides. This table lists the relative abundance and CV for all analytes that could be quantified using either of the three methods, based on a set of 9 replicates. Peak 4a and peak 4b could not be quantified separately using ThermoFisher Chromeleon but was quantified as a singular peak. The individual values for peaks 4a and peak 4b obtained from Waters Empower and HappyTools were summed to compare with ThermoFisher Chromeleon.
(XLSX)

S4 Table Comparison of precision between Waters Empower, ThermoFisher Chromeleon and HappyTools. The below table calculates the fold change of the CVs between Waters Empower, ThermoFisher Chromeleon and HappyTools by dividing the HappyTools CV with either the Waters Empower or ThermoFisher Chromeleon CV. The results show an average fold change improvement of 2.22 (vs. Waters Empower) and 2.26 (vs. ThermoFisher Chromeleon). Peaks 4a and 4b were not used in the HappyTools vs. ThermoFisher Chromeleon comparison because these peaks could not be quantified separately using ThermoFisher Chromeleon.
(XLSX)

S5 Table HappyTools results of total ACPA-IgG quantitation. The relative area of all quantified glycans are displayed in the presented table. The native G1F and G2S2 levels have also been included in the column, which were calculated by summing all glycan peaks that match G1F (GP8 and GP9) or G2S2 (GP21, GP22, GP23 and GP24).
(XLSX)

S6 Table HappyTools results of ACPA-IgG Fab quantitation. The relative area of all quantified glycans are displayed in the presented table. The native G1F and G2S2 levels have also been included in the column, which were calculated by summing all glycan peaks that match G1F (GP8a, GP8b and GP9) or G2S2 (GP21, GP22, GP23 and GP24).
(XLSX)

S7 Table ACPA-IgG Fab glycosylation. The table below lists the calculation of the percentage of Fab glycosylation, where the calculation is (G2S2_Total_ / G2S2_Fab_) / (G1F_Total_ / G1F_Fc_). The data for G1F_Fc_ was derived by glycopeptide analysis and was taken directly from a previously published study [17].
(XLSX)

S1 Data Zip file containing HappyTools and the raw data files. The source code of HappyTools is included in this zip file, together with all the raw chromatograms as exported from ThermoFisher Chromeleon. A visual tutorial and a document demonstrating how to reproduce the results used in this study are also included.
(ZIP)

## References

[1] Varki A. Biological roles of oligosaccharides: all of the theories are correct. Glycobiology. 1993;3: 97–130. doi: 10.1093/glycob/3.2.97 8490246 10.1093/glycob/3.2.97 PMC7108619

[2] Arnold JN, Wormald MR, Sim RB, Rudd PM, Dwek RA. The Impact of Glycosylation on the Biological Function and Structure of Human Immunoglobulins. Annu Rev Immunol. Annual Reviews; 2007;25: 21–50. doi: 10.1146/annurev.immunol.25.022106.141702 17029568 10.1146/annurev.immunol.25.022106.141702

[3] Pučić M, Knežević A, Vidič J, Adamczyk B, Novokmet M, Polašek O, et al High Throughput Isolation and Glycosylation Analysis of IgG–Variability and Heritability of the IgG Glycome in Three Isolated Human Populations. Mol Cell Proteomics. 2011;10 doi: 10.1074/mcp.M111.010090 21653738 10.1074/mcp.M111.010090 PMC3205872

[4] Waters. eXtended performance [Internet]. 2013. http://www.waters.com/webassets/cms/library/docs/720004195en.pdf

[5] Ali I, Al-Othman ZA, Nagae N, Gaitonde VD, Dutta KK. Recent trends in ultra-fast HPLC: New generation superficially porous silica columns. J Sep Sci. 2012;35: 3235–3249. doi: 10.1002/jssc.201200454 23184368 10.1002/jssc.201200454

[6] Soliven A, Foley D, Pereira L, Hua S, Edge T, Ritchie H, et al Improving the performance of narrow-bore HPLC columns using active flow technology. Microchem J. 2014;116: 230–234. https://doi.org/10.1016/j.microc.2014.05.006

[7] Royle L, Campbell MP, Radcliffe CM, White DM, Harvey DJ, Abrahams JL, et al HPLC-based analysis of serum N-glycans on a 96-well plate platform with dedicated database software. Anal Biochem. 2008;376: 1–12. https://doi.org/10.1016/j.ab.2007.12.012 18194658 10.1016/j.ab.2007.12.012

[8] Thomson RI, Gardner RA, Strohfeldt K, Fernandes DL, Stafford GP, Spencer DIR, et al Analysis of Three Epoetin Alpha Products by LC and LC-MS Indicates Differences in Glycosylation Critical Quality Attributes, Including Sialic Acid Content. Anal Chem. American Chemical Society; 2017;89: 6455–6462. doi: 10.1021/acs.analchem.7b00353 28509534 10.1021/acs.analchem.7b00353

[9] Walsh I, O’Flaherty R, Rudd PM. Bioinformatics applications to aid high-throughput glycan profiling. Perspect Sci. 2017;11: 31–39. doi: 10.1016/j.pisc.2016.01.013

[10] Zhao S, Walsh I, Abrahams JL, Royle L, Nguyen-Khuong T, Spencer D, et al GlycoStore: A Database of Retention Properties for Glycan Analysis. Bioinformatics. 2018; bty319-bty319. Available: http://dx.doi.org/10.1093/bioinformatics/bty319 10.1093/bioinformatics/bty319 29897488

[11] Gotz L, Abrahams JL, Mariethoz J, Rudd PM, Karlsson NG, Packer NH, et al GlycoDigest: a tool for the targeted use of exoglycosidase digestions in glycan structure determination. Bioinformatics. 2014;30: 3131–3133. Available: http://dx.doi.org/10.1093/bioinformatics/btu425 25015990 10.1093/bioinformatics/btu425 PMC4609004

[12] Hu Y, Zhou S, Yu C-Y, Tang H, Mechref Y. Automated annotation and quantitation of glycans by liquid chromatography/electrospray ionization mass spectrometric analysis using the MultiGlycan-ESI computational tool. Rapid Commun Mass Spectrom. 2015;29: 135–142. doi: 10.1002/rcm.7093 25462374 10.1002/rcm.7093 PMC4516131

[13] Jansen BC, Reiding KR, Bondt A, Hipgrave Ederveen AL, Palmblad M, Falck D, et al MassyTools: A High-Throughput Targeted Data Processing Tool for Relative Quantitation and Quality Control Developed for Glycomic and Glycoproteomic MALDI-MS. J Proteome Res. American Chemical Society; 2015;14: 5088–5098. doi: 10.1021/acs.jproteome.5b00658 26565759 10.1021/acs.jproteome.5b00658

[14] Jansen BC, Falck D, de Haan N, Hipgrave Ederveen AL, Razdorov G, Lauc G, et al LaCyTools: A Targeted Liquid Chromatography–Mass Spectrometry Data Processing Package for Relative Quantitation of Glycopeptides. J Proteome Res. American Chemical Society; 2016;15: 2198–2210. doi: 10.1021/acs.jproteome.6b00171 27267458 10.1021/acs.jproteome.6b00171

[15] Reiding KR, Ruhaak LR, Uh H-W, el Bouhaddani S, van den Akker EB, Plomp R, et al Human Plasma N-glycosylation as Analyzed by Matrix-Assisted Laser Desorption/Ionization-Fourier Transform Ion Cyclotron Resonance-MS Associates with Markers of Inflammation and Metabolic Health. Mol Cell Proteomics. 2017;16: 228–242. doi: 10.1074/mcp.M116.065250 27932526 10.1074/mcp.M116.065250 PMC5294210

[16] Agakova A, Vučković F, Klarić L, Lauc G, Agakov F. Automated integration of a UPLC glycomic profile In: Lauc G, Wuhrer M, editors. Methods in Molecular Biology. New York, NY: Springer New York; 2017 pp. 217–233. doi: 10.1007/978-1-4939-6493-2_17 10.1007/978-1-4939-6493-2_17 27743370

[17] Hafkenscheid L, Bondt A, Scherer HU, Huizinga TWJ, Wuhrer M, Toes REM, et al Structural Analysis of Variable Domain Glycosylation of Anti-Citrullinated Protein Antibodies in Rheumatoid Arthritis Reveals the Presence of Highly Sialylated Glycans. Mol Cell Proteomics. United States; 2017;16: 278–287. doi: 10.1074/mcp.M116.062919 27956708 10.1074/mcp.M116.062919 PMC5294214

[18] Van Rossum G, Drake Jr FL. Python reference manual. Centrum voor Wiskunde en Informatica Amsterdam; 1995.

[19] van der Walt S, Colbert SC, Varoquaux G. The NumPy array: a structure for efficient numerical computation. Comput Sci Eng. IEEE; 2011;13: 22–30.

[20] Hunter JD. Matplotlib: a 2D Graphics Environment. Comput Sci Eng. IEEE; 2007;9: 90–95. doi: 10.1109/MCSE.2007.55

[21] Oliphant TE. Python for scientific computing. Comput Sci Eng. IEEE; 2007;9.

[22] Foundation TAS. Apache 2.0 License [Internet]. 2004 [cited 24 Oct 2017]. https://www.apache.org/licenses/LICENSE-2.0

[23] Vivó-Truyols G, Torres-Lapasió JR, van Nederkassel AM, Vander Heyden Y, Massart DL. Automatic program for peak detection and deconvolution of multi-overlapped chromatographic signals: Part I: Peak detection. J Chromatogr A. 2005;1096: 133–145. https://doi.org/10.1016/j.chroma.2005.03.092 16301076 10.1016/j.chroma.2005.03.092

[24] Yu T, Peng H. Quantification and deconvolution of asymmetric LC-MS peaks using the bi-Gaussian mixture model and statistical model selection. BMC Bioinformatics. 2010;11: 559 doi: 10.1186/1471-2105-11-559 21073736 10.1186/1471-2105-11-559 PMC2993707

[25] Nevedomskaya E, Derks R, Deelder AM, Mayboroda OA, Palmblad M. Alignment of capillary electrophoresis–mass spectrometry datasets using accurate mass information. Anal Bioanal Chem. 2009;395: 2527 doi: 10.1007/s00216-009-3166-1 19826795 10.1007/s00216-009-3166-1

[26] Katajamaa M, Oresic M. Data processing for mass spectrometry-based metabolomics. J Chromatogr A. 2007;1158 doi: 10.1016/j.chroma.2007.04.021 17466315 10.1016/j.chroma.2007.04.021

[27] Schumaker LL. Spline Functions: Computational Methods. SIAM; 2015.

[28] Reusch D, Haberger M, Maier B, Maier M, Kloseck R, Zimmermann B, et al Comparison of methods for the analysis of therapeutic immunoglobulin G Fc-glycosylation profiles—Part 1: Separation-based methods. MAbs. Taylor & Francis; 2015;7: 167–179. doi: 10.4161/19420862.2014.986000 25524468 10.4161/19420862.2014.986000 PMC4623496

[29] Longden H. Linking data to electronic records. Qual Assur J. John Wiley & Sons, Ltd.; 2003;7: 84–91. doi: 10.1002/qaj.217

[30] Waters. The Role of Empower Chromatography Data Software in Assisting with Electronic Records Regulation Compliance. 2018.

