# Supplementary figures and images for: HappyTools: A software for high-throughput HPLC data processing and quantitation
Source: PLoS One. 2018 Jul 6;13(7):e0200280. doi: 10.1371/journal.pone.0200280 (PMC6034860; doi:10.1371/journal.pone.0200280)

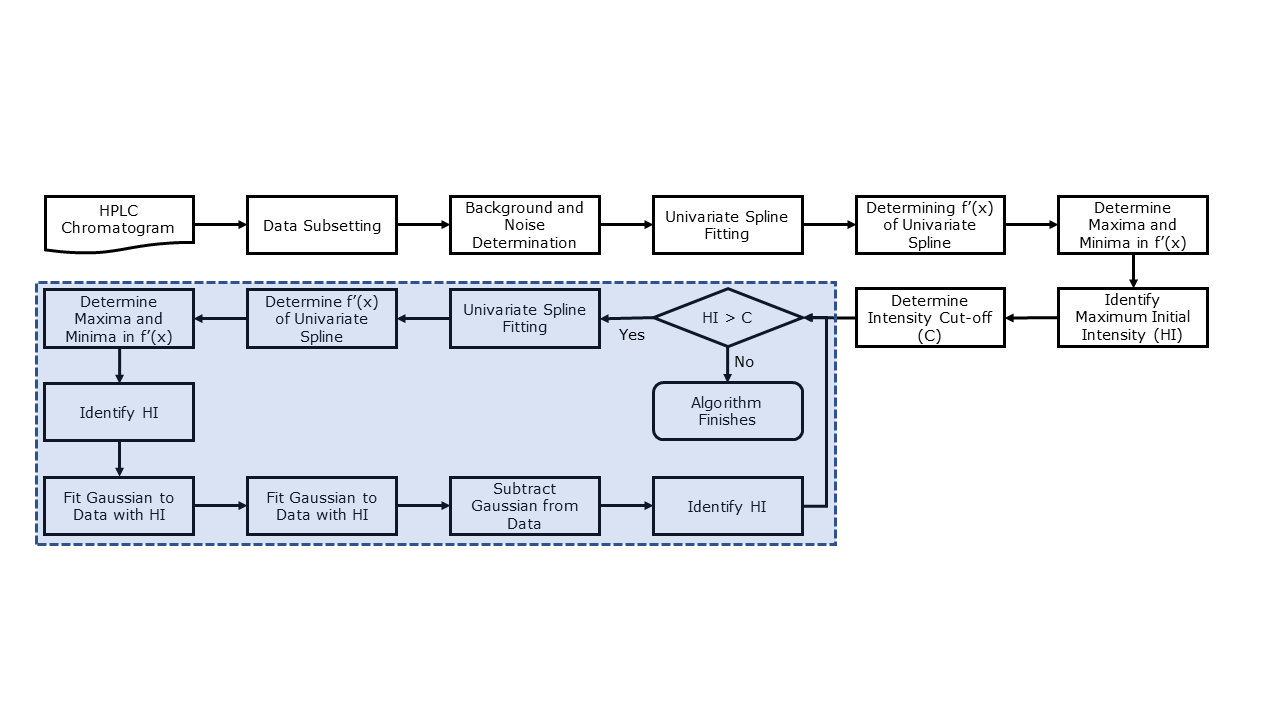

Supplement: S1 Fig — The algorithm first creates a subset of the data, based on the user specified region of interest to ensure that artefacts are not examined by the algorithm. Subsequently, the background and noise are determined which will be used as a baseline for later Gaussian fitting steps. The 1st order derivative is determined of a univariate spline that has been fitted to the data subset. The borders for each peak in the chromatogram is then determined by derivatizing the univariate spline and identifying where the local maxima and minima are of f’(x). The highest intensity data point of all peaks in the user specified region of interest is used to determine the intensity cut-off (e.g. 1% of the initial highest intensity). The main part of the algorithm is then repeated until the highest intensity data point is no longer above the intensity cut-off, and within each loop the borders of all remaining peaks are first determined by using a new univariate spline and it’s derivative. Subsequently, a Gaussian is fitted to the data that yields the highest intensity data point, after which the Gaussian is subtracted from the data. (TIFF) [file pone.0200280.s001.tiff]

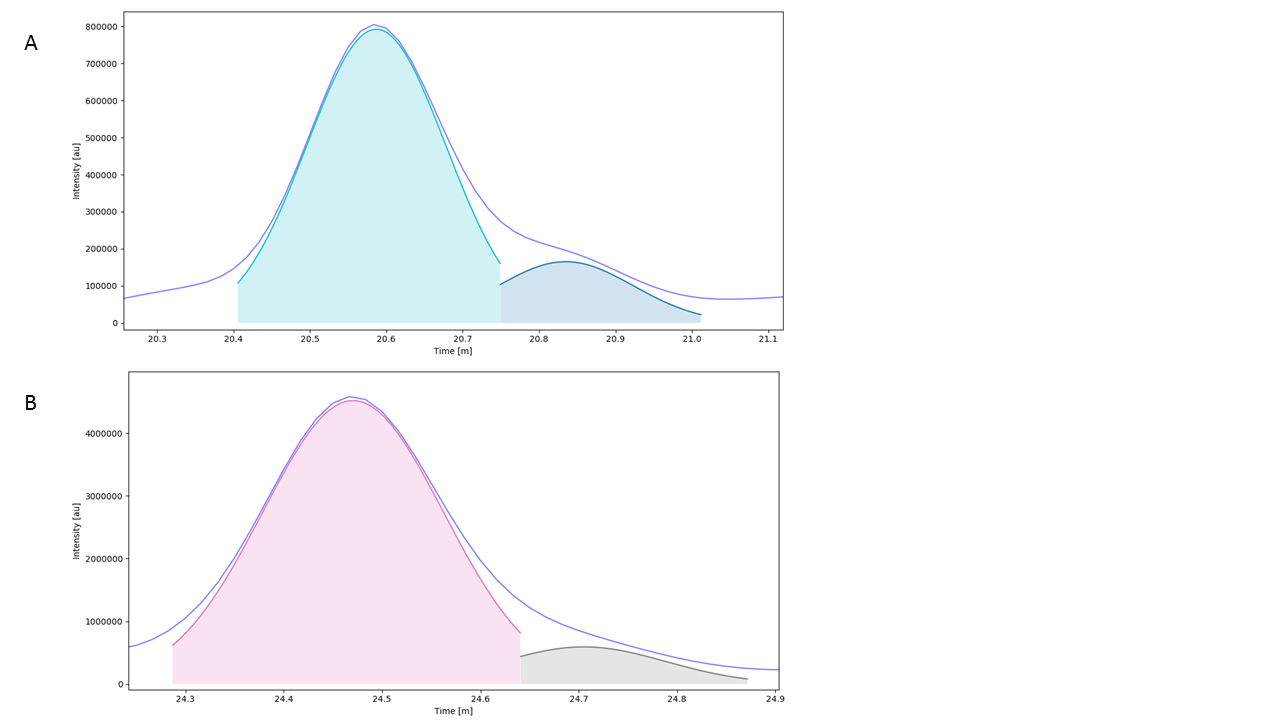

Supplement: S2 Fig — HappyTools uses a Gaussian function to identify chromatographic peaks, which can result in a single non-Gaussian peak being resolved as multiple peaks. (A) Two partially overlapping that can be confidently resolved using HappyTools, (B) A non-Gaussian peak or two partially overlapping Gaussian peaks, which is resolved as two separate peaks by HappyTools. These images were taken directly from HappyTools, after disabling the legend. (TIFF) [file pone.0200280.s002.tiff]

Detail view: 3

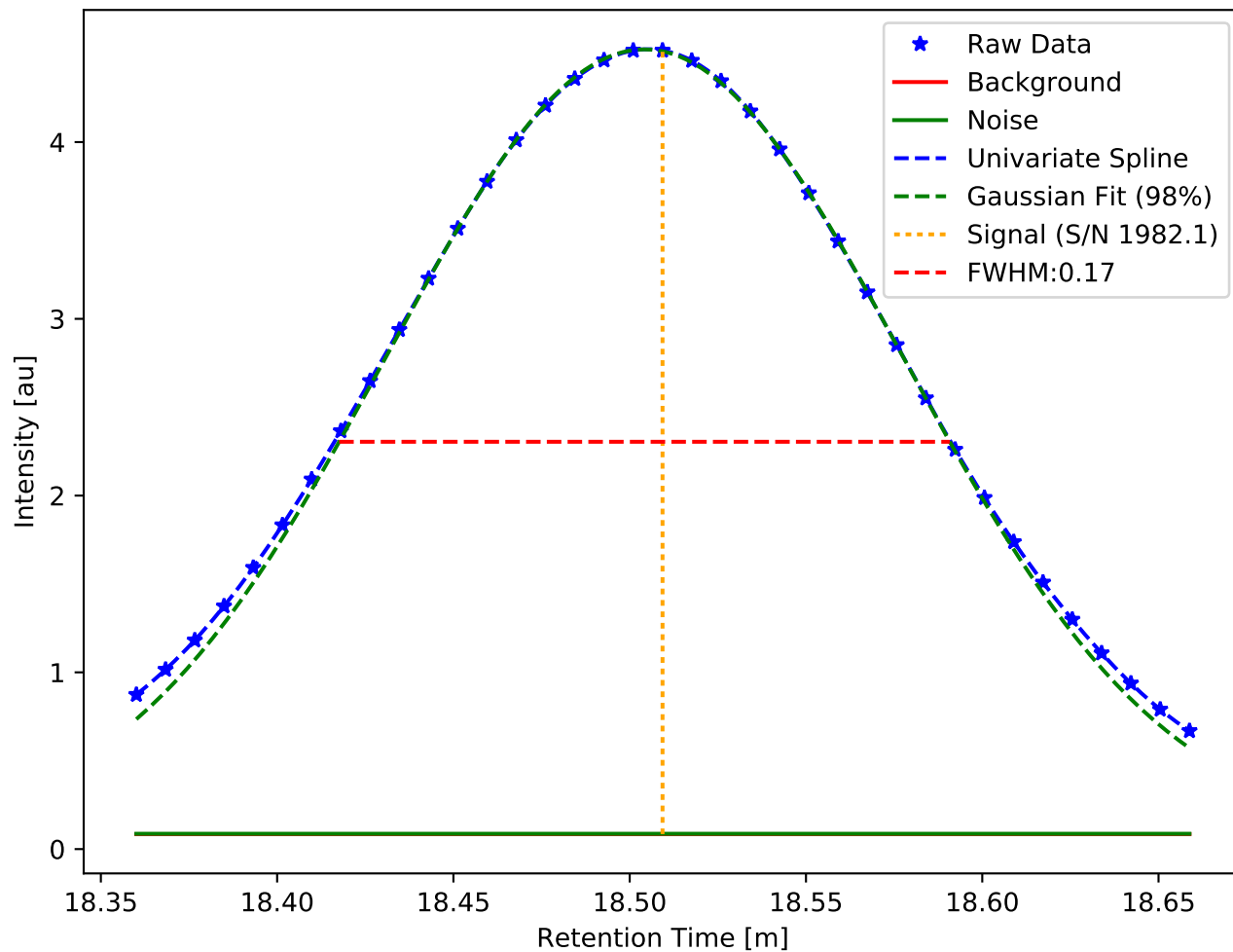

Supplement: S3 Fig — This figure illustrates how the raw data is used to fit both a univariate spline and a Gaussian peak. The univariate spline is used to determine the centre of the experimental peak, which is used to determine the signal-to-noise ratio. The Gaussian fit is used to determine how much of the experimental peak area can be explained by an underlying Gaussian peak, which is the Gaussian peak Quality (GPQ). (PDF) [file pone.0200280.s003.pdf]

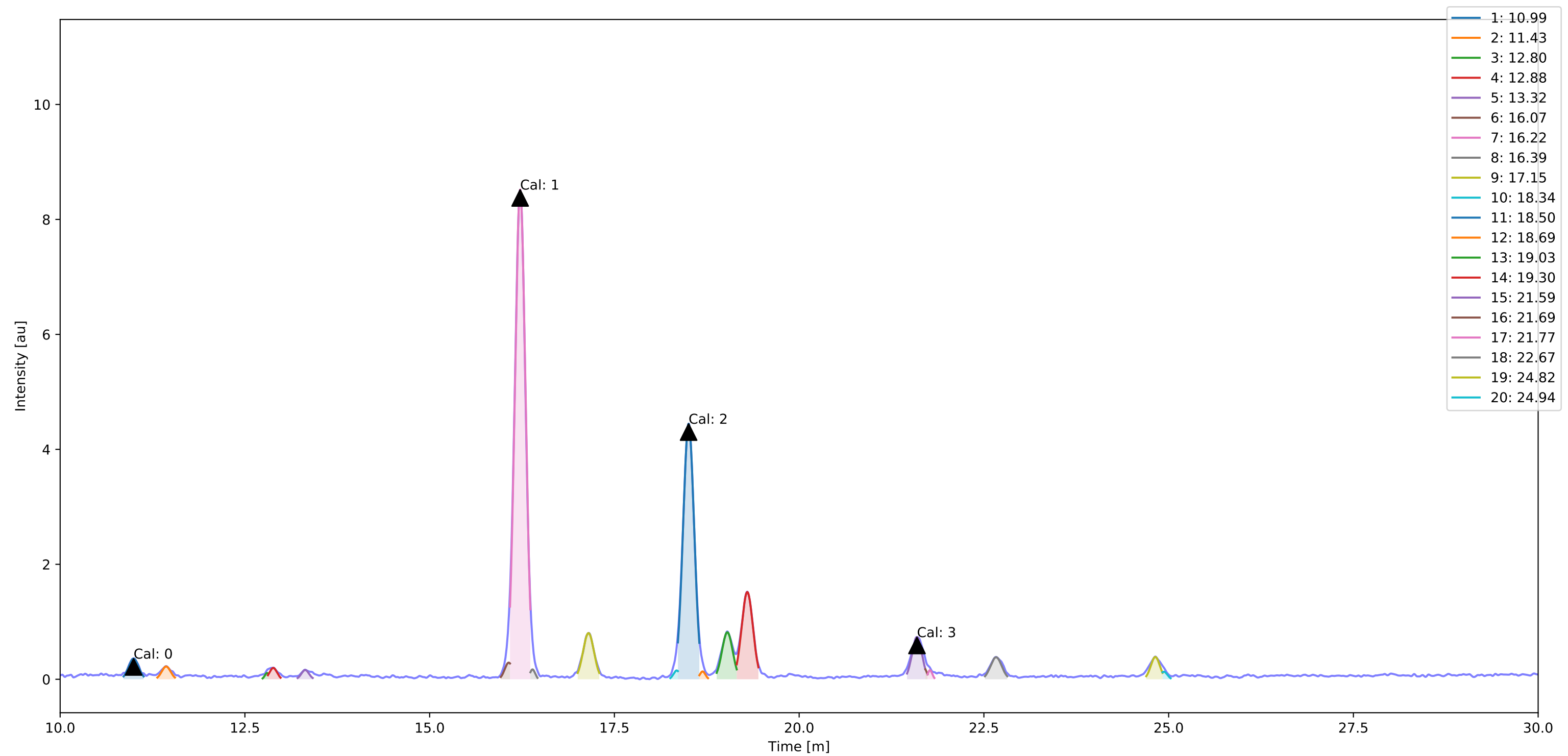

Supplement: S4 Fig — A total of 20 peaks was detected using HappyTools’ peak detection functionality between 10.0 and 30.0 min using a peak detection threshold of 1%. The displayed peak width was selected to be 2σ. However, several of the detected peaks are caused by either overlapping peaks or non-Gaussian peak shapes. Manual curation of the automatically detected peaks reduces the number to 13–15. (PDF) [file pone.0200280.s004.pdf]

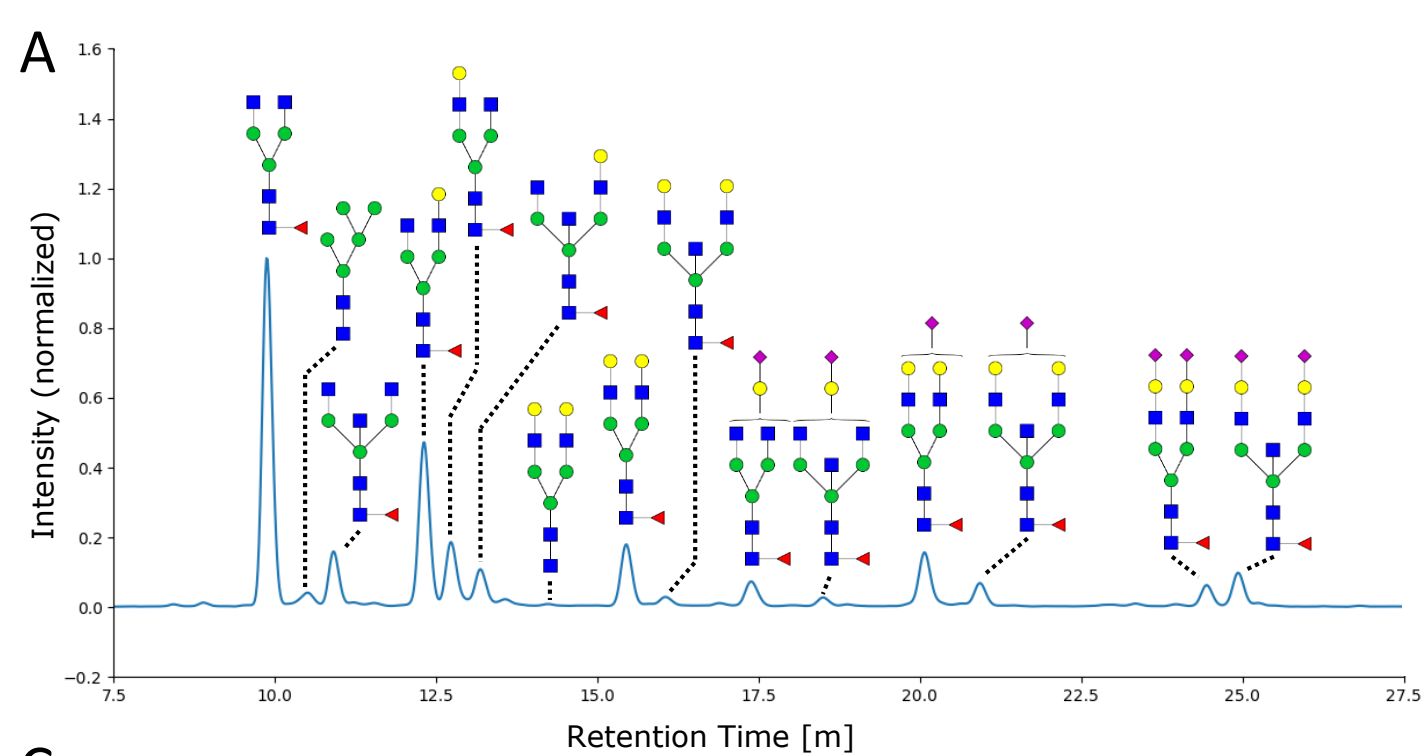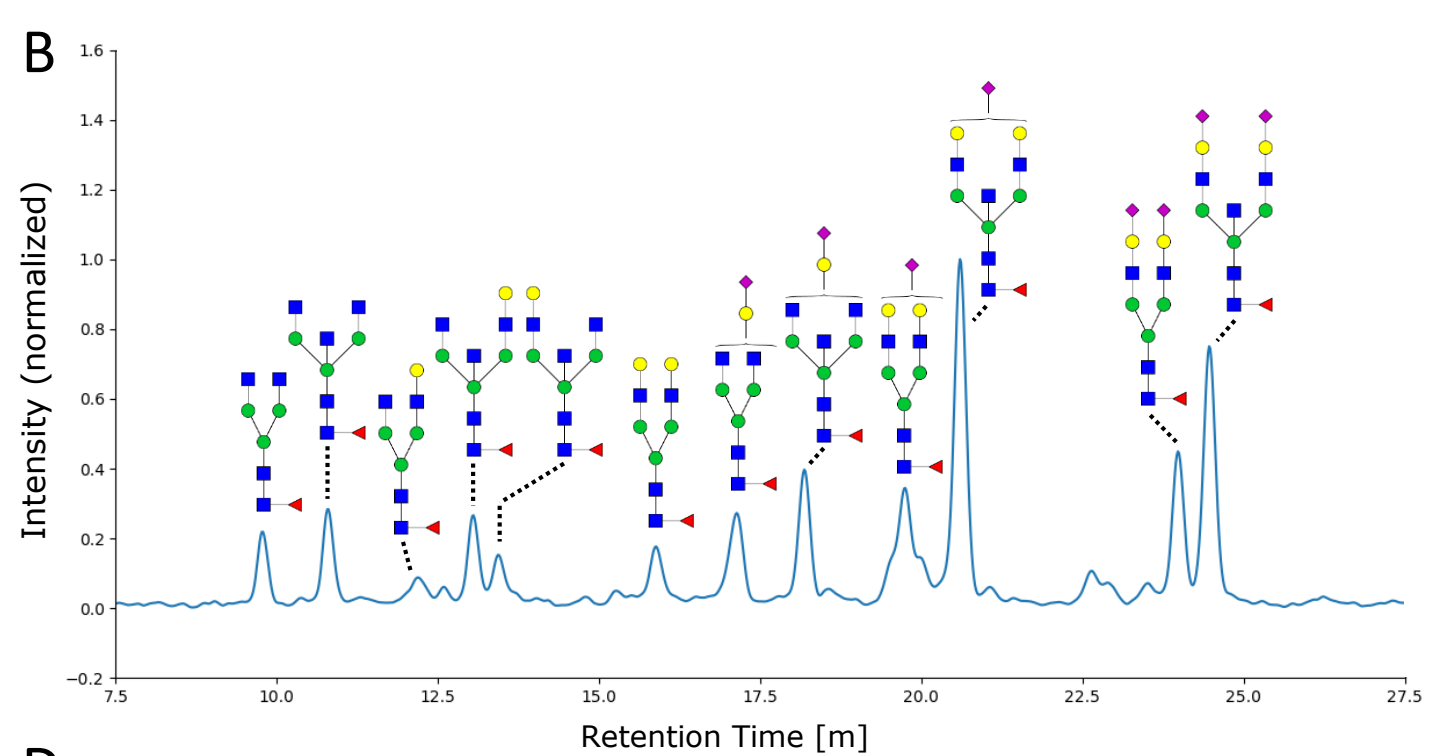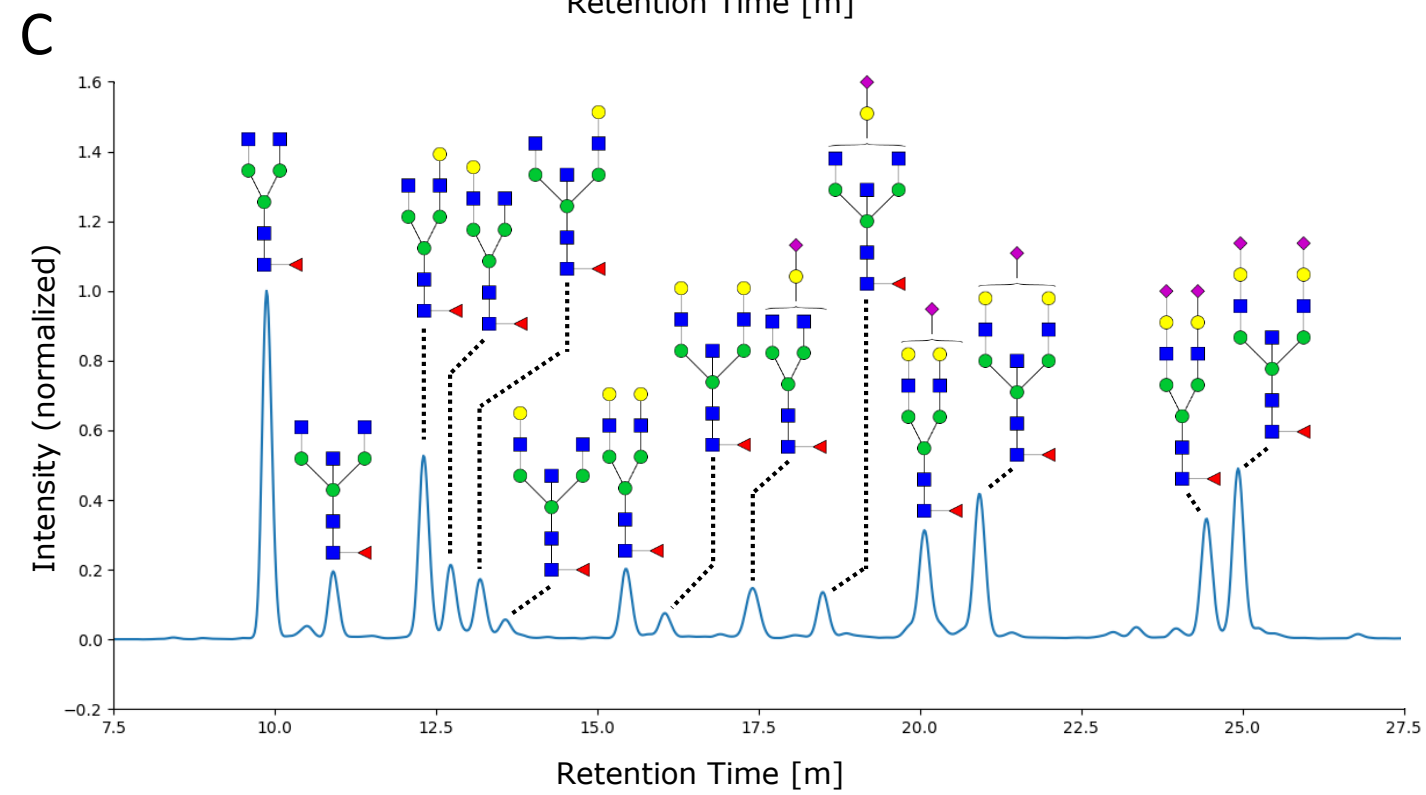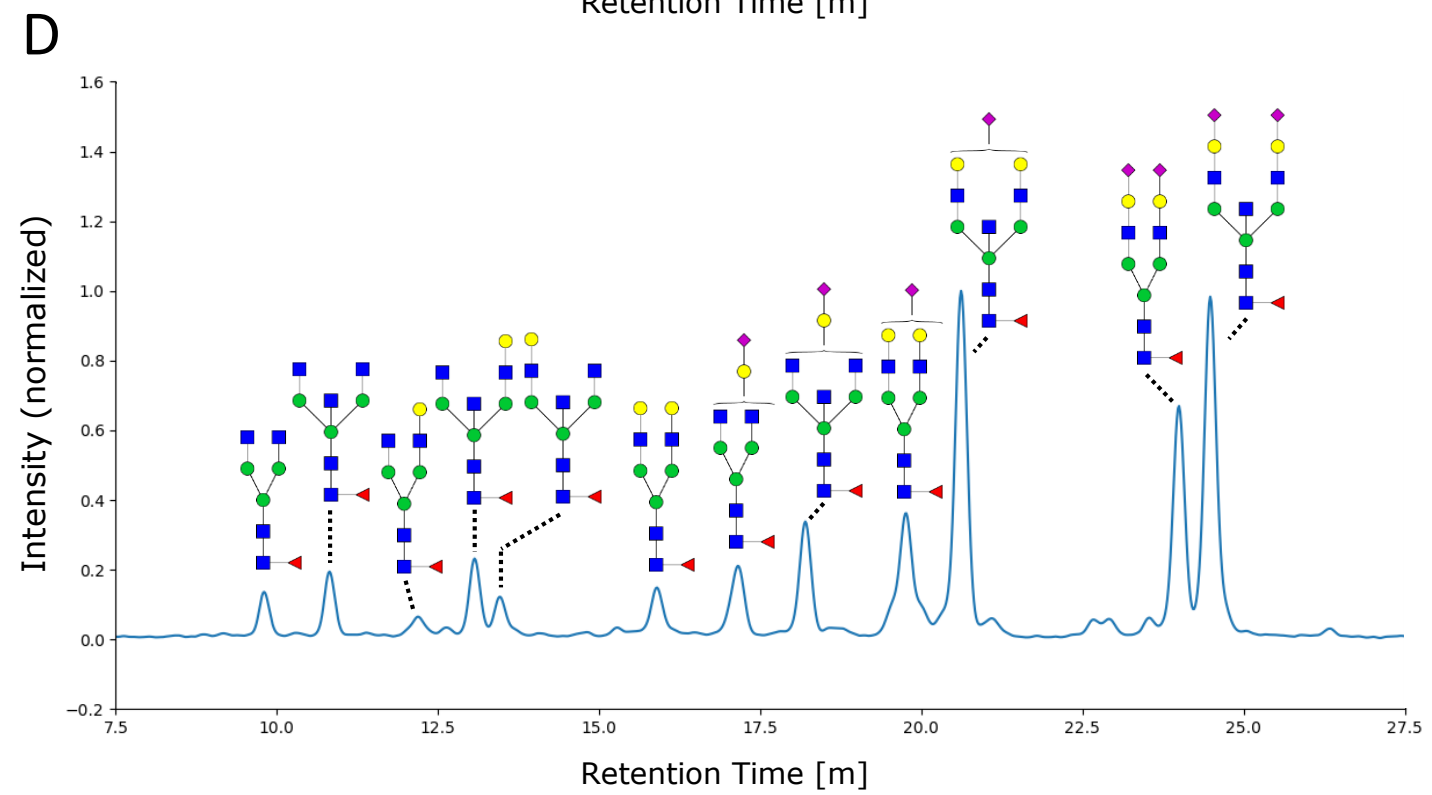

Supplement: S5 Fig — (A) IgG chromatogram, (B) IgG-Fab chromatogram, (C) ACPA-IgG chromatogram and (D) ACPA-IgG Fab chromatogram of patient 4. All chromatograms have been normalised to the highest peak between 10 and 60 minutes. The chromatograms have been plotted using the ‘Normalized Batch Plot’ functionality of HappyTools. The displayed glycan structures are based on the original publication that first measured and described these samples [17]. (PDF) [file pone.0200280.s005.pdf]

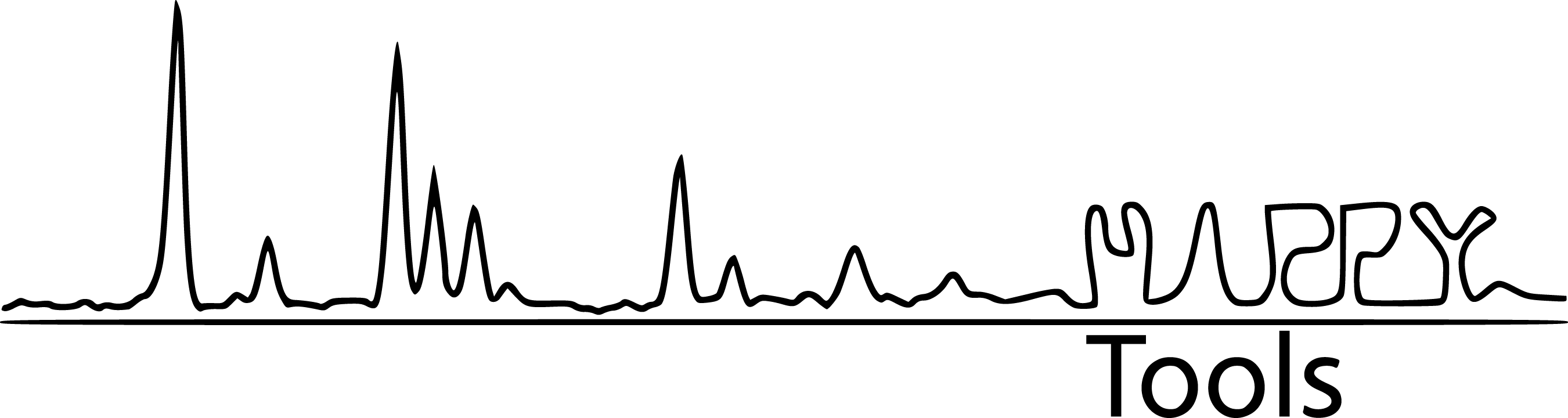

Supplement: S1 Data — The source code of HappyTools is included in this zip file, together with all the raw chromatograms as exported from ThermoFisher Chromeleon. A visual tutorial and a document demonstrating how to reproduce the results used in this study are also included. (ZIP) [file pone.0200280.s013.zip › Data/HappyTools (Source)/ui/UI.png]
