# Supplementary material for: HappyTools: A software for high-throughput HPLC data processing and quantitation
Source: PLoS One. 2018 Jul 6;13(7):e0200280. doi: 10.1371/journal.pone.0200280 (PMC6034860; doi:10.1371/journal.pone.0200280)
Supplement: S1 Data — The source code of HappyTools is included in this zip file, together with all the raw chromatograms as exported from ThermoFisher Chromeleon. A visual tutorial and a document demonstrating how to reproduce the results used in this study are also included. (ZIP) [file pone.0200280.s013.zip › Data/HappyTools - How to reproduce publication results.pdf]

Biopharmaceutical Data

1. Navigate to the “Data\Biopharmaceutical” folder and copy the HappyTools.ini file (which contains the settings, used to process this data) to the “Data\HappyTools (Compiled)” folder.
2. Run HappyTools by selecting the “HappyTools.exe” file in the “Data\HappyTools (Compiled)” folder.
3. Click the “Batch Process” button.
4. Click the “Calibration File” button of the “Batch Process” window.
5. Navigate to the “Data\Biopharmaceutical” folder, select the “20180516 IgG1_Calibrants.ref” file and click “Ok”.
6. Click the “Analyte File” button of the “Batch Process” window.
7. Navigate to the “Data\Biopharmaceutical” folder, select the “20180516 IgG1_Analytes.ref” file and click “Ok”.
8. Click the “Batch Directory” button of the “Batch Process” window.
9. Select the “Data\Biopharmaceutical” folder and click “Ok”.
10. Click the “Output Options” button of the “Batch Process” window.
11. Click the “Select All” button and click “Ok”.
12. Click the “Run” button.
13. The results will appear in the “Data\Biopharmaceutical” folder (both a summary file and individual PDF reports for each chromatogram).

Clinical Data

1. Navigate to the “Data\Clinical\ACPA-IgG Fab” folder and copy the HappyTools.ini file (which contains the settings, used to process this data) to the “Data\HappyTools (Compiled)” folder.
2. Run HappyTools by selecting the “HappyTools.exe” file in the “Data\HappyTools (Compiled)” folder.
3. Click the “Batch Process” button.
4. Click the “Calibration File” button of the “Batch Process” window.
5. Navigate to the “Data\Clinical\ACPA-IgG Fab” folder, select the “fabcallist.ref” file and click “Ok”.
6. Click the “Analyte File” button of the “Batch Process” window.
7. Navigate to the “Data Clinical\ACPA-IgG Fab” folder, select the “fabpeaklist.ref” file and click “Ok”.
8. Click the “Batch Directory” button of the “Batch Process” window.
9. Select the “Data\Clinical\ACPA-IgG Fab” folder and click “Ok”.
10. Click the “Output Options” button of the “Batch Process” window.
11. Click the “Select All” button and click “Ok”.
12. Click the “Run” button.
13. The results will appear in the “Data\Clinical\ACPA-IgG Fab” folder (both a summary file and individual PDF reports for each chromatogram).
14. Repeat the above steps for using the “Data\Clinical\ACPA-IgG Part 1” and “Data\Clinical\ACPA-IgG Part 2” folders, with the following changes:
    a. For the “Calibration File” step select “Total_Part1_callist.ref” for “Data\Clinical\ACPA-IgG Part 1” and “Total_Part2_Callist.ref” for “Data\Clinical\ACPA-IgG Part 2”.
    b. For the “Analyte File” step select “Total_Part1_peaklist.ref” for “Data\Clinical\ACPA-IgG Part 1” and “Total_Part2_peaklist.ref” for “Data\Clinical\ACPA-IgG Part 2.
