# Supplementary material for: HappyTools: A software for high-throughput HPLC data processing and quantitation
Source: PLoS One. 2018 Jul 6;13(7):e0200280. doi: 10.1371/journal.pone.0200280 (PMC6034860; doi:10.1371/journal.pone.0200280)
Supplement: S1 Data — The source code of HappyTools is included in this zip file, together with all the raw chromatograms as exported from ThermoFisher Chromeleon. A visual tutorial and a document demonstrating how to reproduce the results used in this study are also included. (ZIP) [file pone.0200280.s013.zip › Data/HappyTools v0.0.2 Tutorial.pdf]

HappyTools v0.0.2 Basic Tutorial

Bas Cornelis Jansen - 23rd May 2018

This document describes how HappyTools can be used to process a set of 9 replicate measurements of V-Tag labelled tryptic IgG glycopeptides, that were used in the PLoS one submission. The tutorial will present all the steps that must be performed and includes a brief explanation of all the HappyTools settings towards the end of this document.

Python Installation

HappyTools can be run using the Python source code or using the Windows binary (that is released with every major release). This section can be skipped if one is using the ready to go Windows binary.

HappyTools requires Python 2.7, NumPy, SciPy, Matplotlib and Tkinter to run from the source code. We suggest the use of a pre-packaged Python distribution that comes with many of the commonly used libraries pre-installed such as Anaconda Python or Python(x,y). HappyTools can be run by starting the HappyTools.py program after Python has been installed. Note; on some windows computers it is required to associate ".py" files with python. This can be done by double clicking the "HappyTools.py" file which should then show the following window:

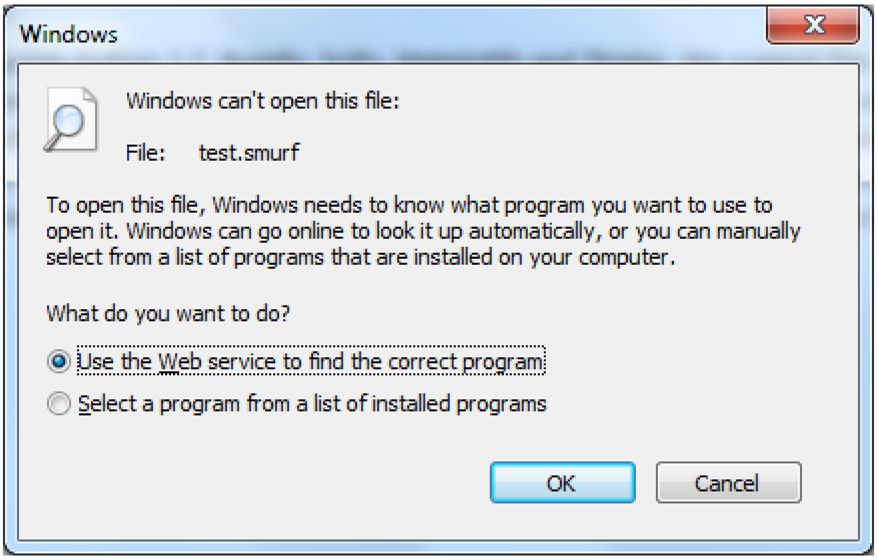

Select the "Select a program from a list of installed programs", which should show the following screen:

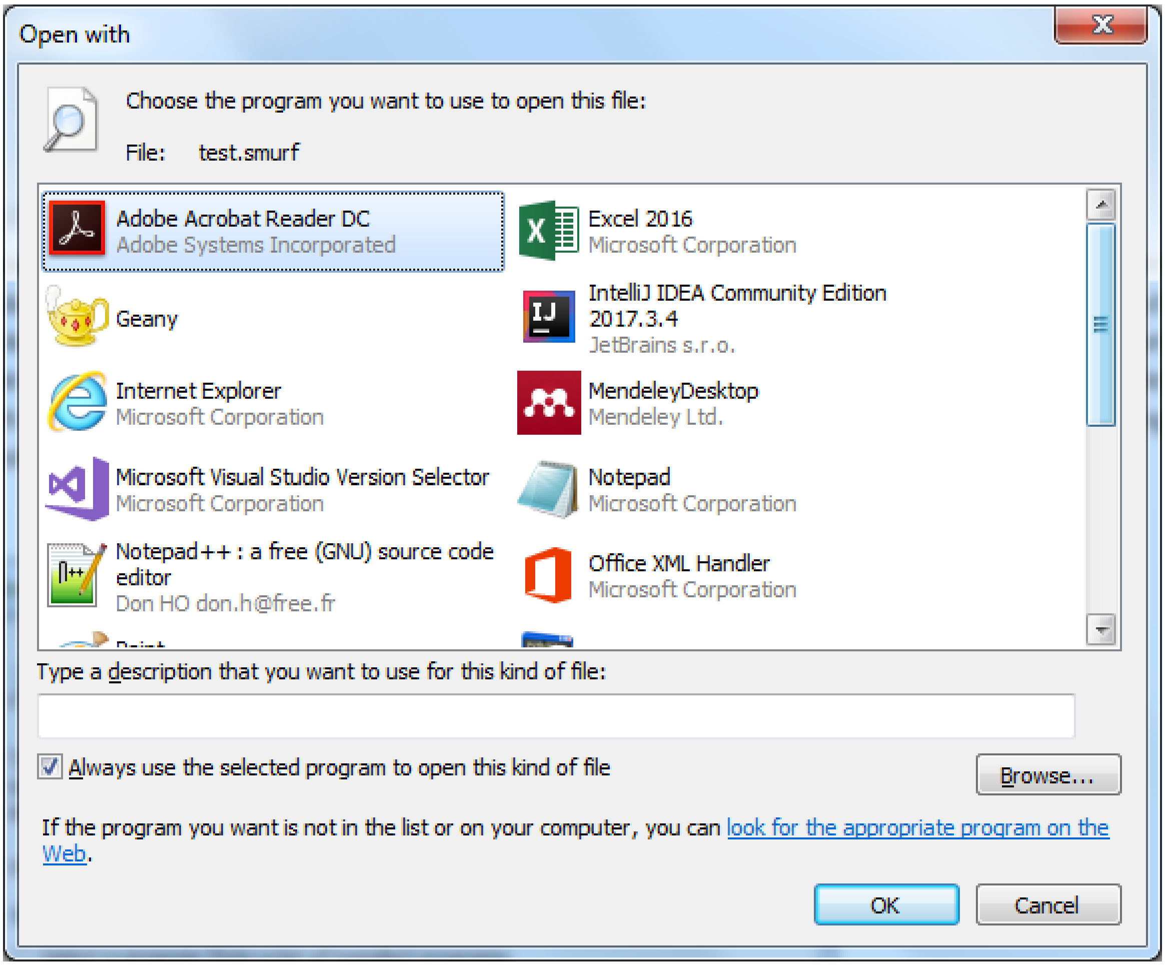

Click the ‘Browse’ button in the lower right corner and navigate to the location where Python has been installed and select the “Python.exe” file as shown in the below screenshot:

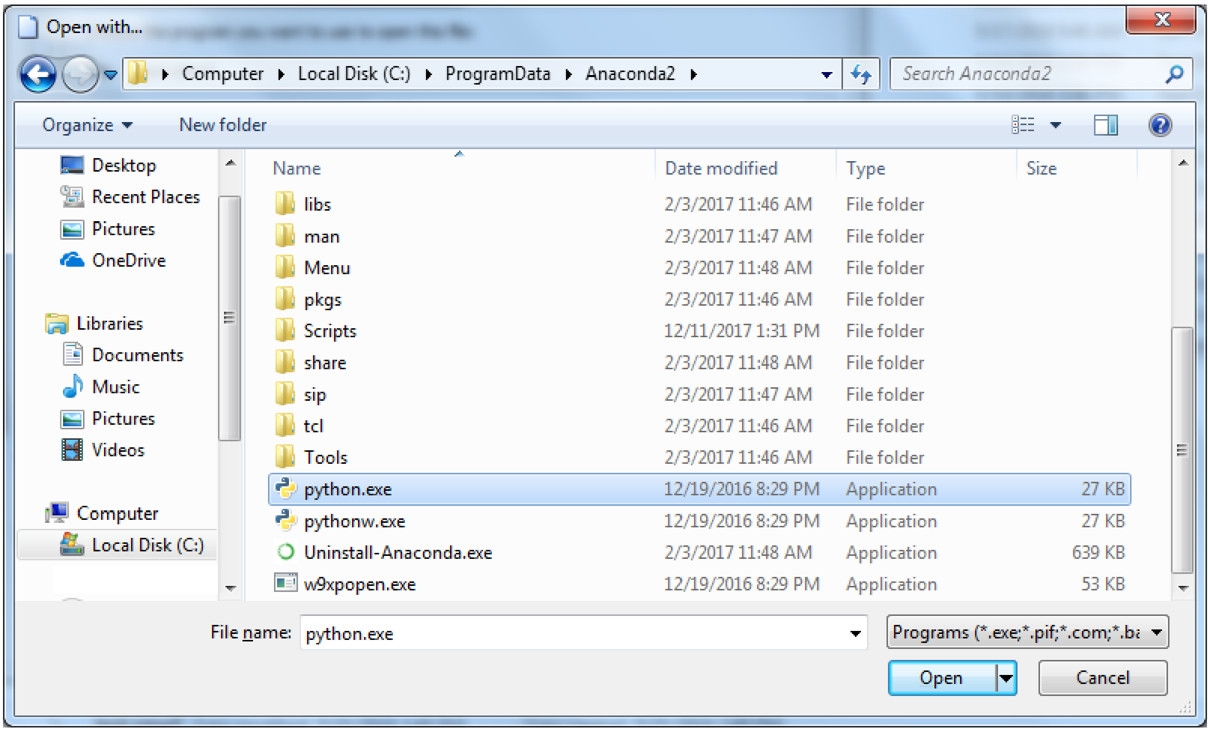

Alternatively, HappyTools can be run by using the ready to go windows binary file, by running the HappyTools.exe file that is included in major releases.

Demonstration using supplied trial data

This section will illustrate how to perform HappyTools processing, using a set of 9 replicate measurements of V-Tag labelled tryptic IgG glycopeptides, that were used in the PLoS one submission. The demonstration will assume that the user knows nothing about the sample yet.

Peak Detection

Open HappyTools and open a chromatogram by going "File > Open Chromatogram", navigate to the "test" folder and select a chromatogram (this demo will use IgG Vtag 1_ACQUITY FLR ChA.txt). This should produce the following screen:

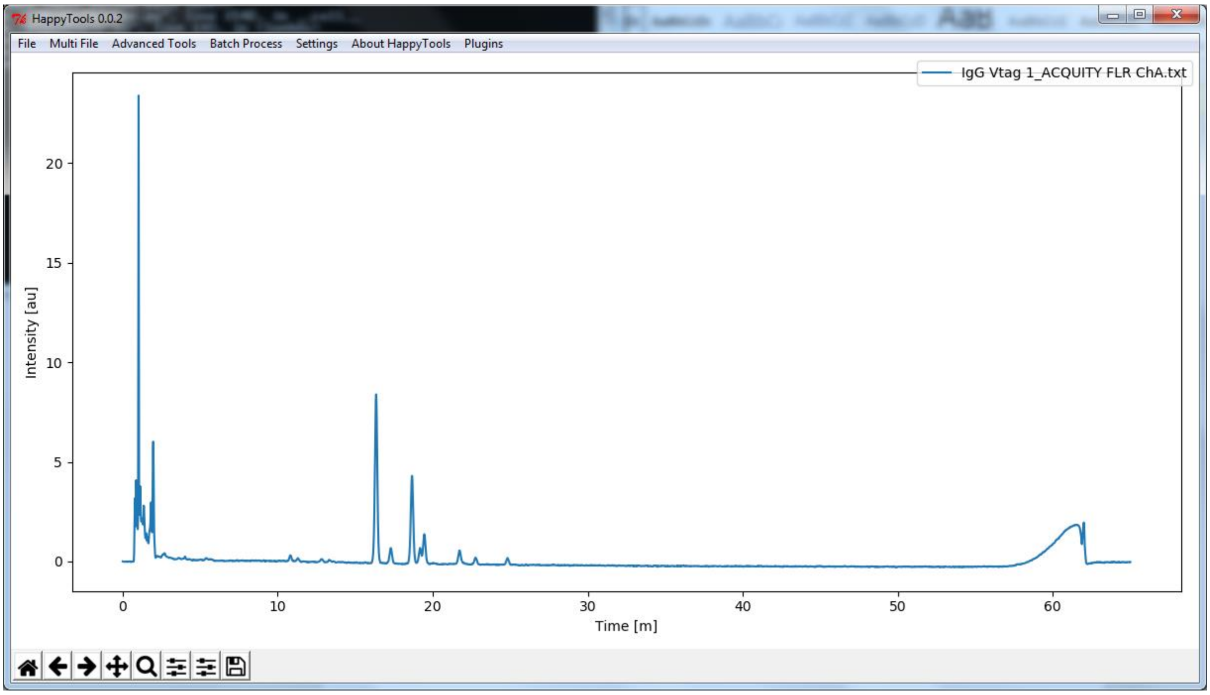

Next click "Settings" and adjust the "Start" and "End" time parameters so that only the region of interest will be examined for automated peak detection, baseline correction and other processing steps (for the demo, we will use 15 to 30 minutes). Furthermore, the user can select if HappyTools will use sigma (σ) or the full width at half maximum (FWHM) for peak width, as shown in the below screenshot of the settings window:

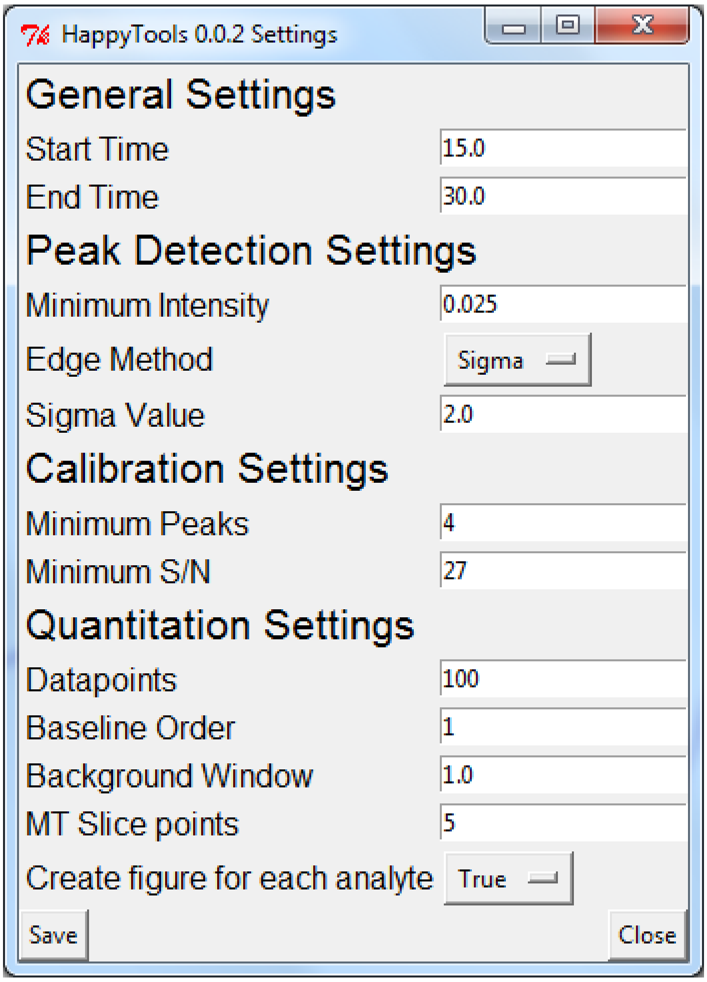

Click "Save" and then "Close" once you are happy with the settings. Next, there are a few steps that might need to be performed on some chromatograms, namely baseline correction and smoothing. Click the magnifying glass icon and select a small region around the baseline, which should yield the following screen:

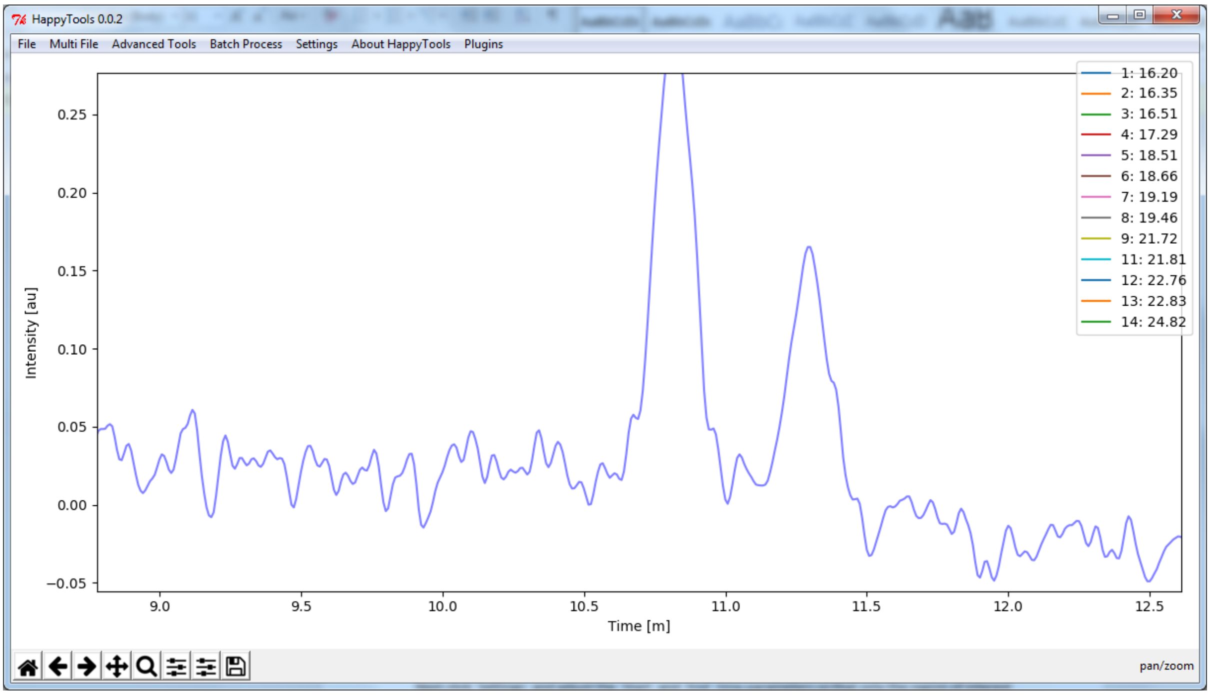

Here we can see that the intensity does go below 0, therefore we should perform a baseline correction prior to any further processing. This can be done via the "File > Baseline Correction" button, which should then show the following window:

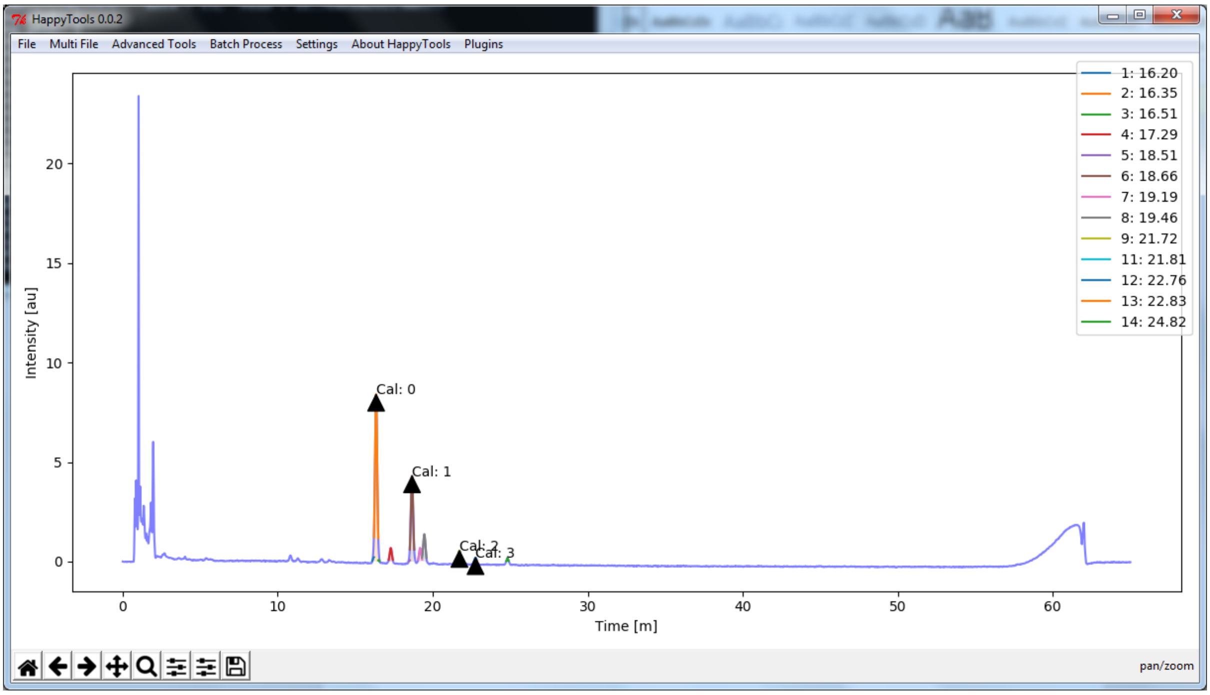

We can zoom in on the region of interest either via the magnifying glass or by manually defining the X- and Y-axes (the first slider button), if we change the X-axis to 15-30 minutes and the Y-axis to 0-10 au, we should see the following image:

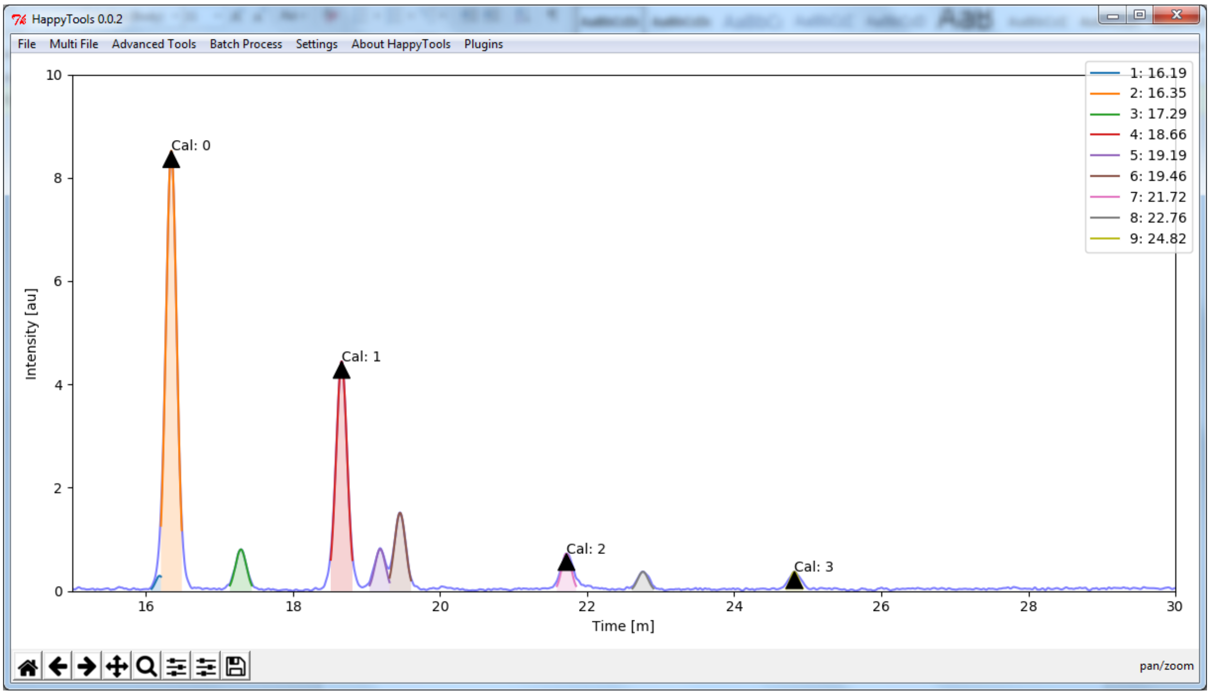

This shows that HappyTools has detected 9 peaks, which included all the clearly visible peaks. We can save the suggested list of calibrant peaks and analytes by selecting “Advanced Tools > Save Calibrants” and “Advanced Tools > Save Annotation”, we will call these files “auto_calibrants.ref” and “auto_analytes.ref”.

The selection of calibrants has a significant impact on the latter quantitation, ideally calibrants are roughly equally spaced throughout the entire time region of interest and number around 4 or 5. The automatic determination of calibrants marks the four maximum peaks in the four time regions that are acquired by dividing the entire time region in four equal length sub-regions.

In the above figure, it is not possible to say if the first peak is an actual peak or just the effect of some fronting. Therefore, we will remove this peak in the next step by hand. This can be done by by opening the “auto_analytes.ref” file in Excel (or notepad++) and then removing the first line, as shown in the below screenshot:

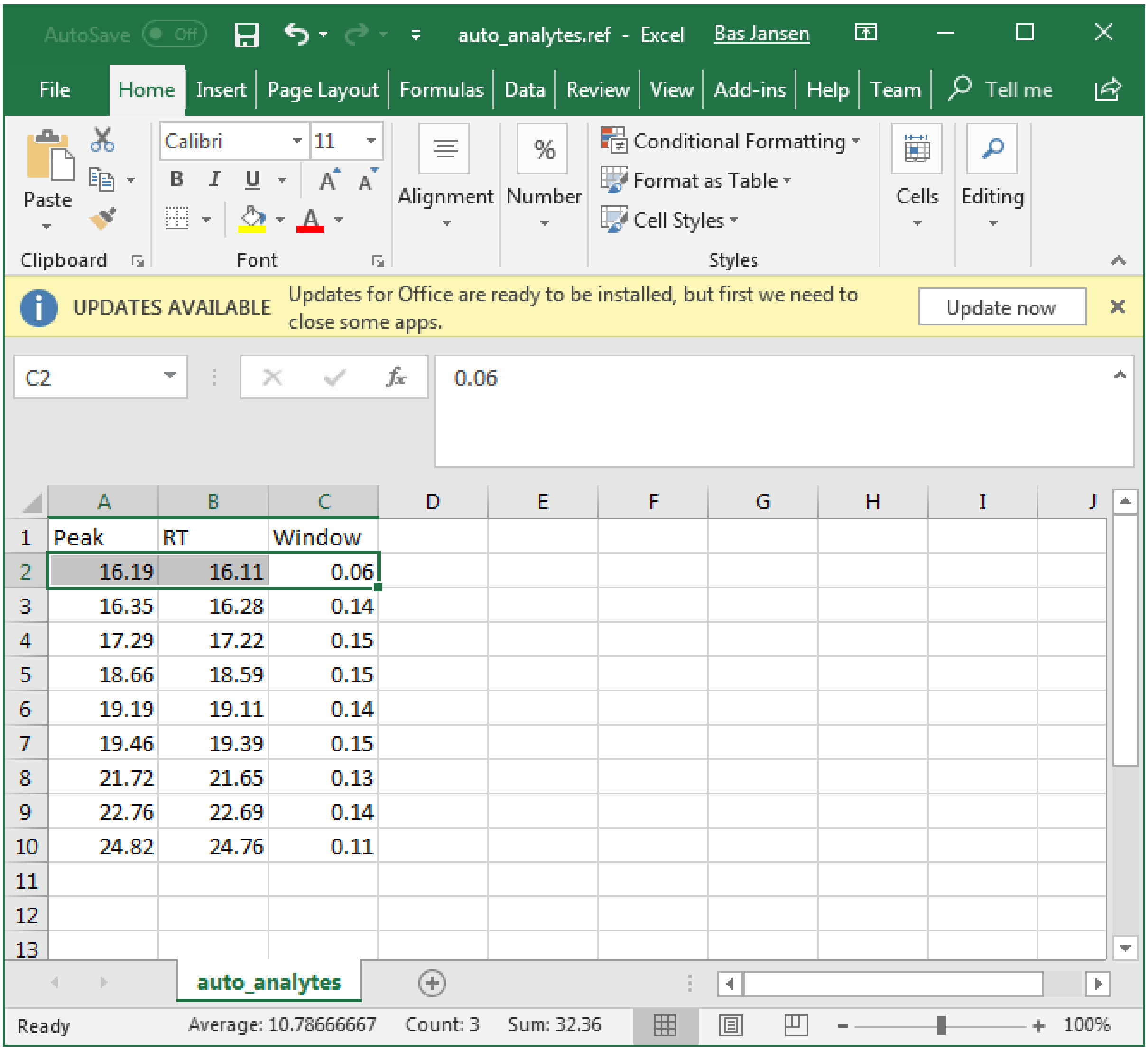

The line that we will delete is marked, make sure that one uses the option to delete an entire line by right clicking on the 2 (on the left side of the screen). The file can now be saved back to the disk, by the standard save function of Excel. The user can also decide to add an additional peak in this file or to adjust the retention time of a peak if desired, please note that it is essential that for each peak that will be quantified it is required to have a name, a retention time value for the peak maximum and a retention time window for quantitation. It is likewise possible to adjust the calibration list, with one key difference that the retention time window in the calibration list refers to the time range that HappyTools will examine, to find the peak maximum.

Batch Processing

We will now perform a “Batch Process”, which means that HappyTools will calibrate all chromatograms based on the list of automatically detected calibrants prior to quantifying the automatically detected peaks in all chromatograms. This is achieved by clicking the “Batch Process” button, which should show the following screen:

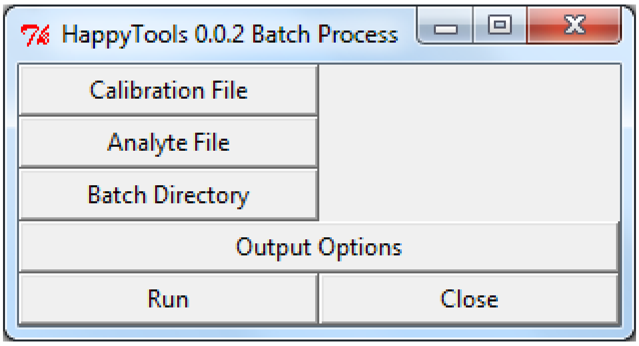

This screen has four buttons that must be set, by clicking on them and selecting the corresponding file or directory. First, click the “Calibration File” button and select the “auto_cal.ref” file. Next, click the “Analyte File” button and select the “auto_analytes.ref” file. The location of the raw data can be selected by clicking the “Batch Directory” button and selecting the “test” folder. The user can choose which outputs they want to have in the final summary file by clicking the “Output Options” button, and selecting the relevant outputs (we suggest to always extract all parameters). The screen should now look as follows:

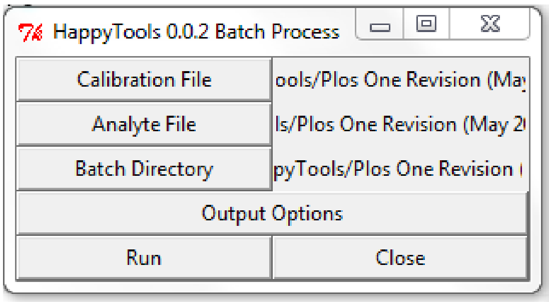

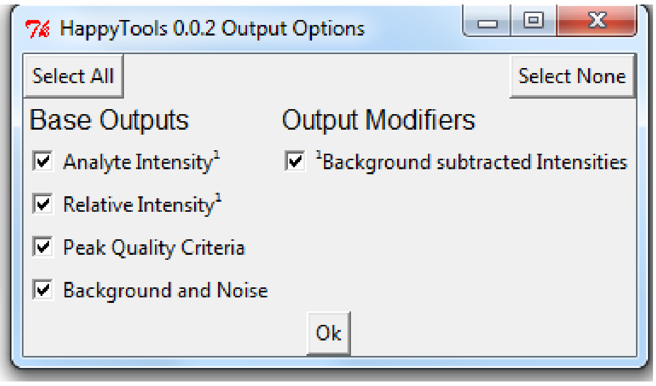

Cal: 1

Close the “Output Options” screen by clicking the “Ok” button. Lastly, the “Batch Process” will begin by clicking the “Run” button, which should show a progress bar as follows:

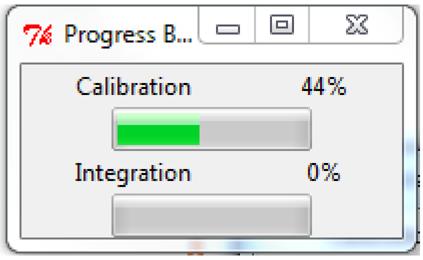

A pop-up window will appear once HappyTools is finished with the “Batch Process”, that looks as follows:

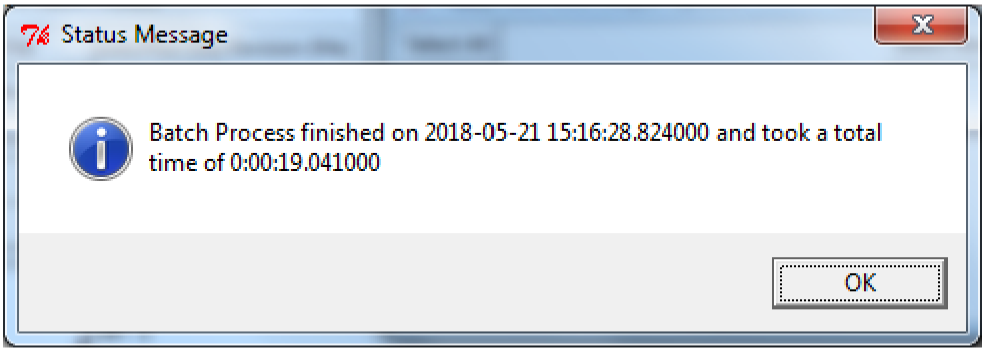

The results will now be available in the “test” folder, and should be stored in a file with a name that contains the term “summary.results” that is pre-fixed with the time that HappyTools was run. This file is a tab-separated file and can be opened with Excel, which should then look as follows:

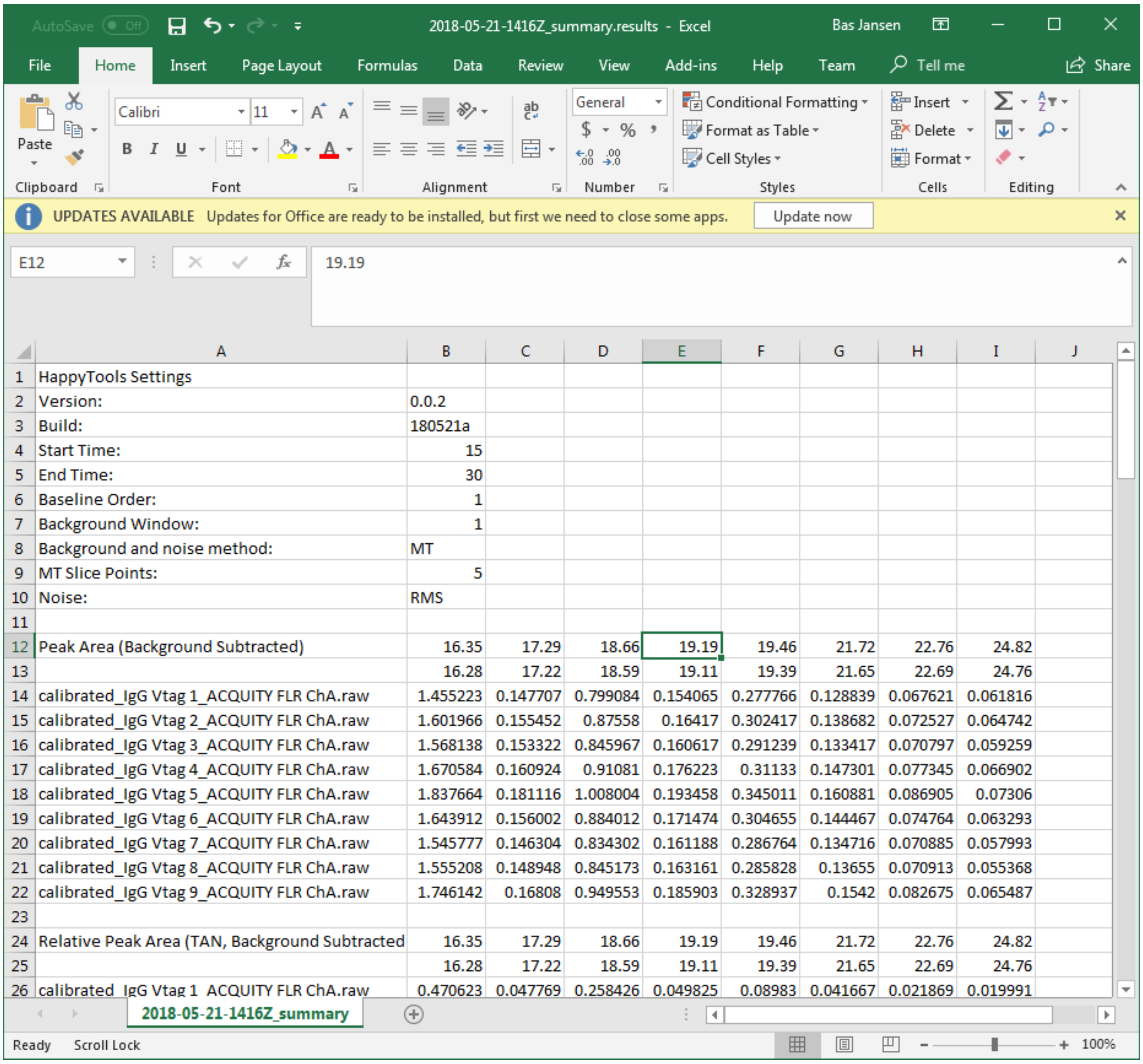

	A	B	C	D	E	F	G	H	I	J
1	HappyTools Settings									
2	Version:	0.0.2								
3	Build:	180521a								
4	Start Time:	15								
5	End Time:	30								
6	Baseline Order:	1								
7	Background Window:	1								
8	Background and noise method:	MT								
9	MT Slice Points:	5								
10	Noise:	RMS								
11										
12	Peak Area (Background Subtracted)	16.35	17.29	18.66	19.19	19.46	21.72	22.76	24.82	
13		16.28	17.22	18.59	19.11	19.39	21.65	22.69	24.76	
14	calibrated_IgG Vtag 1_ACQUITY FLR ChA.raw	1.455223	0.147707	0.799084	0.154065	0.277766	0.128839	0.067621	0.061816	
15	calibrated_IgG Vtag 2_ACQUITY FLR ChA.raw	1.601966	0.155452	0.87558	0.16417	0.302417	0.138682	0.072527	0.064742	
16	calibrated_IgG Vtag 3_ACQUITY FLR ChA.raw	1.568138	0.153322	0.845967	0.160617	0.291239	0.133417	0.070797	0.059259	
17	calibrated_IgG Vtag 4_ACQUITY FLR ChA.raw	1.670584	0.160924	0.91081	0.176223	0.31133	0.147301	0.077345	0.066902	
18	calibrated_IgG Vtag 5_ACQUITY FLR ChA.raw	1.837664	0.181116	1.008004	0.193458	0.345011	0.160881	0.086905	0.07306	
19	calibrated_IgG Vtag 6_ACQUITY FLR ChA.raw	1.643912	0.156002	0.884012	0.171474	0.304655	0.144467	0.074764	0.063293	
20	calibrated_IgG Vtag 7_ACQUITY FLR ChA.raw	1.545777	0.146304	0.834302	0.161188	0.286764	0.134716	0.070885	0.057993	
21	calibrated_IgG Vtag 8_ACQUITY FLR ChA.raw	1.555208	0.148948	0.845173	0.163161	0.285828	0.13655	0.070913	0.055368	
22	calibrated_IgG Vtag 9_ACQUITY FLR ChA.raw	1.746142	0.16808	0.949553	0.185903	0.328937	0.1542	0.082675	0.065487	
23										
24	Relative Peak Area (TAN, Background Subtracted	16.35	17.29	18.66	19.19	19.46	21.72	22.76	24.82	
25		16.28	17.22	18.59	19.11	19.39	21.65	22.69	24.76	
26	calibrated_IgG Vtag 1_ACQUITY FLR ChA.raw	0.470623	0.047769	0.258426	0.049825	0.08983	0.041667	0.021869	0.019991	

This file is constructed so that all relevant variables are listed at the top, followed by all the desired outputs that were requested by the user. For example, in the above image the block “Peak Area

(Background Subtracted)" is the first block, followed by "Relative Peak Area (TAN, Background Subtracted)". Furthermore, HappyTools will have created a PDF report for each file that was quantified (unless the Create Figure setting was changed to False), that contain both an overview image and a detailed image of each peak, below is an example of a detail image of a peak:

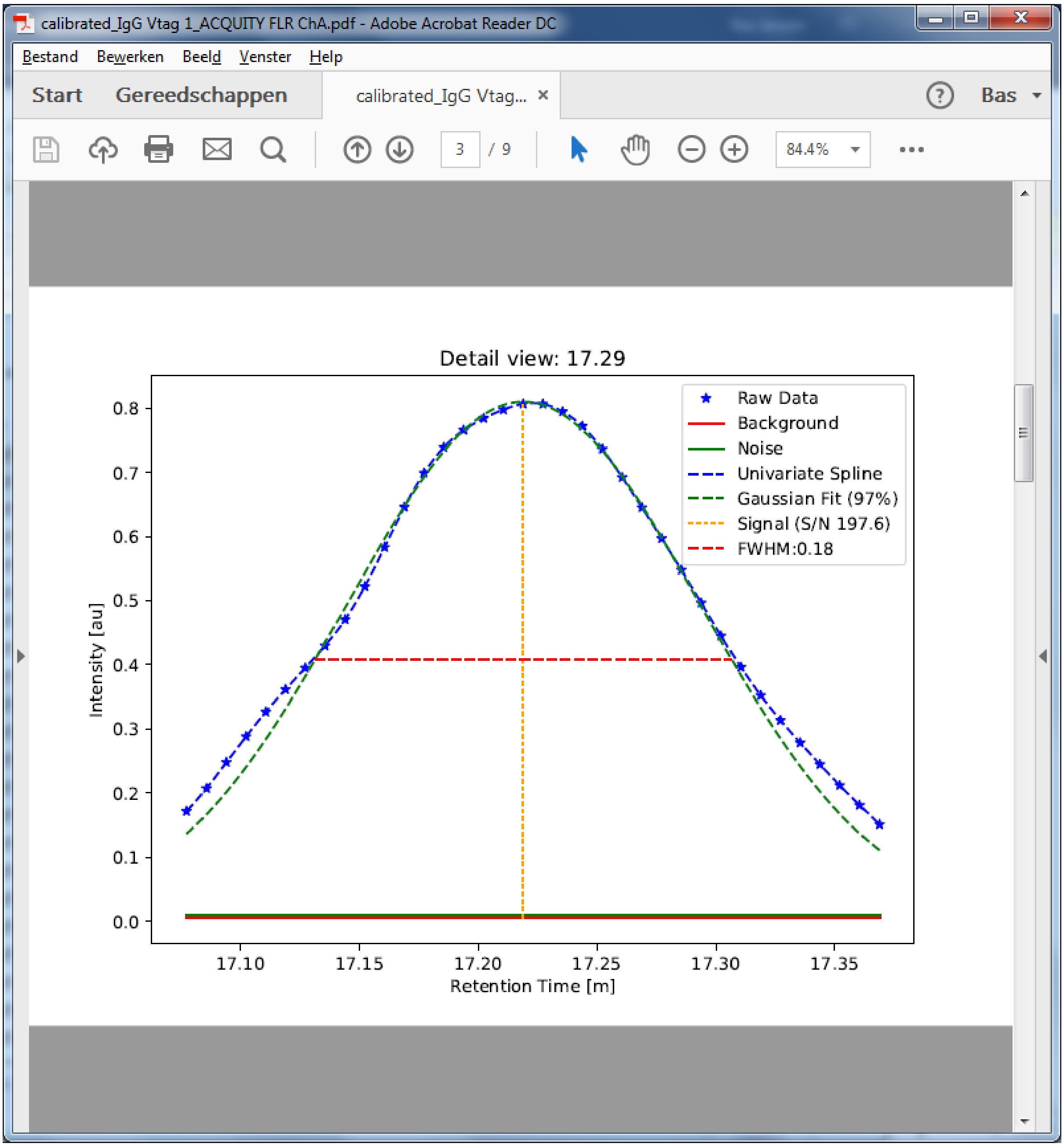

Frequently Asked Questions

Q1. I have selected an "Analyte File" and a "Batch Folder" but HappyTools says it has finished processing without actually quantifying any of my files, why did this occur?

A1. HappyTools is designed to only quantify calibrated files, the program expects that any chromatogram has "calibrated_" as a prefix. A way to circumvent this is by manually renaming the chromatogram and adding "calibrated_" in front of the filename. Alternatively, if one is using the source code then this can be changed by editing the "libs/batchFunctions.py" file. The line "INTEGRATION_FILETYPES = ["calibrated*.txt"]" should then be changed to "INTEGRATION_FILETYPES = ["*.txt"]" (if using "txt" files")

Q2. The retention time of my peaks vary a lot between runs, can I still use HappyTools to calibrate and quantify my data?

A2. Yes! That is exactly the type of problem that HappyTools is meant to address. The only condition is that there are some peaks (4 or 5) that are clear local maxima within a given time region. We suggest to first calibrate all chromatograms using a wider Δt_r prior to visually verifying if the calibration is satisfactory (using the batch plot functionality), prior to performing quantitation.
